# Polygenic Propensity for Longevity, *APOE*-ε4 Status, Dementia Diagnosis, and Risk for Cause-Specific Mortality: A Large Population-Based Longitudinal Study of Older Adults

**DOI:** 10.1093/gerona/glad168

**Published:** 2023-07-12

**Authors:** Olesya Ajnakina, Diana Shamsutdinova, Daniel Stahl, Andrew Steptoe

**Affiliations:** Department of Biostatistics & Health Informatics, Institute of Psychiatry, Psychology and Neuroscience, King’s College London, London, UK; Department of Behavioural Science and Health, Institute of Epidemiology and Health Care, University College London, London, UK; Department of Biostatistics & Health Informatics, Institute of Psychiatry, Psychology and Neuroscience, King’s College London, London, UK; Department of Biostatistics & Health Informatics, Institute of Psychiatry, Psychology and Neuroscience, King’s College London, London, UK; Department of Behavioural Science and Health, Institute of Epidemiology and Health Care, University College London, London, UK

**Keywords:** *APOE*-ε4, Longevity, Mediation, Mortality, Polygenic risk

## Abstract

To deepen the understanding of genetic mechanisms influencing mortality risk, we investigated the impact of genetic predisposition to longevity and *APOE*-ε4, on all-cause mortality and specific causes of mortality. We further investigated the mediating effects of dementia on these relationships. Using data on 7 131 adults aged ≥50 years (mean = 64.7 years, standard deviation [*SD*] = 9.5) from the English Longitudinal Study of Aging, genetic predisposition to longevity was calculated using the polygenic score approach (PGS_longevity_). *APOE*-ε4 status was defined according to the absence or presence of ε4 alleles. The causes of death were ascertained from the National Health Service central register, which was classified into cardiovascular diseases, cancers, respiratory illness, and all other causes of mortality. Of the entire sample, 1 234 (17.3%) died during an average 10-year follow-up. One-*SD* increase in PGS_longevity_ was associated with a reduced risk for all-cause mortality (hazard ratio [HR] = 0.93, 95% confidence interval [CI]: 0.88–0.98, *p* = .010) and mortalities due to other causes (HR = 0.81, 95% CI: 0.71–0.93, *p* = .002) in the following 10 years. In gender-stratified analyses, *APOE*-ε4 status was associated with a reduced risk for all-cause mortality and mortalities related to cancers in women. Mediation analyses estimated that the percent excess risk of *APOE*-ε4 on other causes of mortality risk explained by the dementia diagnosis was 24%, which increased to 34% when the sample was restricted to adults who were aged ≤75 years old. To reduce the mortality rate in adults who are aged ≥50 years old, it is essential to prevent dementia onset in the general population.

In high-income countries, the leading causes of death among older adults are noncommunicable diseases, such as cancers (1), cardiovascular diseases (2,3), and respiratory-related diseases (1). Dementia is another important factor contributing to high mortalities; indeed, of all deaths registered in 2019 in England and Wales, 12.5% were due to dementia diagnosis [4]. Worryingly, the mortality rates due to dementia have been gradually increasing over the last 10 years (4), which may be attributed to population aging. Even though mortality risks are influenced by a combination of factors, including changes in health care systems, and emergent health threats (1), because each individual has a unique biology (5), it is imperative to consider underlying genetic factors when understanding mechanisms underlying mortality risk.

Based on twin studies, the average heritability of longevity in adults has been estimated to be approximately 25%, which tends to increase linearly with age (6,7). The realization that the human lifespan is influenced by genetic factors ignited a search for genetic markers of large effects on longevity through a systematic testing of the entire human genome (8–10); this approach was termed as genome-wide association studies (GWASs). In longevity research, GWAS entails comparisons of the frequency of genetic variants between very long-lived persons and the average population (8–10). Although GWASs for longevity yielded a considerable sparsity of novel locus conferring survival (11), there is one exception.

Indeed, through numerous GWASs, apolipoprotein E (*APOE*) gene located on chromosome 19, which is a liver polypeptide that serves as a ligand for the low-density lipoprotein (LDL) receptor (12), emerged as a major genetic determinant influencing longevity (11,13–15) explaining 12%–17% of the variation in mortality in people ≥65 years (16). In fact, *APOE* is the only gene that meets the criteria for genes with a population-level impact on mortality (17,18). Specifically, the frequencies of *APOE* genotypes, namely *APOE-*ε2, *APOE*-ε3, and *APOE*-ε4, vary in different ages and populations (19). Of the 3 polymorphic forms of *APOE*, carriers of *APOE*-ε4, presumably because of its role in downregulating hepatic LDL receptors (20,21), are at a higher risk of mortality. The evidence further highlights that *APOE*-ε4 carriers are at elevated risk of cause-specific mortality including cardiovascular diseases (20,21), and ischemic heart disease (22), among other mortality causes (23). Because *APOE*-ε4 is also a robust predictor of dementia diagnosis, including Alzheimer’s disease (24–26), it is, however, unknown if the link between all-cause mortality, cause-specific mortality, and *APOE*-ε4 is mediated by dementia diagnosis in older adults.

Further building on the results from GWASs, the polygenic score (PGSs) approach emerged as a quantitative metric of an individual’s inherited risk based on the cumulative impact of many common markers of small effects scattered across the entire genome (27). PGS incorporates genome-wide genetic variation into a single, quantitative measure that can be used to assess genetic susceptibility to an outcome or a trait. This approach showed that a higher polygenetic predisposition to longevity is linearly associated with the human lifespan (28,29). Nonetheless, it is not known if a polygenic predisposition to longevity reduces the risk of all-cause mortality as well as its specific causes in the general population of older adults.

Using a large population-representative cohort of older adults, the aims of the study were twofold. First, we investigated if *APOE*-ε4 status, and polygenic predisposition to longevity, independently from one another was associated with all-cause mortality, and cause-specific mortality, in the following 10 years. Second, we investigated the extent to which the potential relationships of *APOE*-ε4 status with all-cause mortality, and cause-specific mortality were mediated by dementia diagnosis. Here, the mediation hypothesis was that *APOE*-ε4 presence increases the risk of all-cause mortality, and cause-specific mortality, via dementia diagnosis.

## Method

### Study Population

We used data from the English Longitudinal Study of Aging (ELSA), which is an ongoing large, multidisciplinary study of a nationally representative sample of the English population aged ≥50 years (30). The ELSA study started in 2002–2003 (Wave 1) with participants recruited from the Health Survey for England, which was designed to monitor the health of the general population, who were then followed up every 2 years. The ELSA sample is periodically refreshed with younger participants to ensure that the full age spectrum is maintained (30). As the blood (for genetic data) were collected by nurses during a home visit at Wave 2 (2004–2005) for the core members who started at Wave 1, and Wave 4 (2008–2009) for the participants joining the study at Wave 4 through the refreshment sample, the data from these waves formed our baseline. Ethical approval for each of the ELSA waves was granted by the National Research Ethics Service (London Multicentre Research Ethics Committee). All participants gave informed consent.

### Measures

#### Outcome

The outcome was all-cause mortality that occurred from baseline till the end of Wave 8 (2016–17). The date and causes of death were ascertained from the National Health Service central register that captures all deaths occurring in the United Kingdom. Specific causes of mortality were defined using the International Statistical Classification of Diseases and Related Health Problems, Tenth Revision (ICD-10), which were grouped into 4 categories: (1) cardiovascular disease (CVD; coronary artery disease, heart at- tack, and angina pectoris); (2) respiratory-related diseases (emphysema, chronic bronchitis, and chronic obstructive pulmonary disease); (3) cancers; and (4) all other causes, which were also likely to encompass deaths due to dementia (31,32). Survival time was defined as the period from baseline when all ELSA participants were alive to the date when an ELSA participant was reported to have died during the follow-up period. For those who did not die during follow-up, the survival time was calculated using the period spanning from baseline until the end of Wave 8 (2016–17). Because observation periods of >10 years were only available for a very small portion of the sample (Supplementary Figure 1 and Supplementary Table 1) resulting in diminished power for estimating cause-specific mortality risks, in the main association analyses we restricted observation period to ≤10 years in the entire cohort (mean = 9.1, *SD* = 2.0, median = 10.0, 65 222.9 person-years). Therefore, when reporting the results from the main association analyses, we referred to the follow-up period as “10 years.”

#### Ascertainment of dementia cases

Dementia was ascertained during the follow-up period using a physician-made diagnosis of dementia or Alzheimer’s disease (AD). If ELSA participants were unable to respond to the main interview themselves, the 16 items IQCODE was administered to an informant (family member or long-term caregiver), who knew the respondent very well. A threshold of ≥3.38 or more on the IQCODE was used to define dementia (33–35) with high sensitivity (0.84) and specificity (0.86) in the present study. This approach to identifying dementia incidence, including AD, has been widely used in population-based cohorts reinforcing its validity (36–39). The group comparisons in sociodemographic characteristics between participants with and without the dementia diagnosis are shown in Supplementary Table 2.

### Genetic Data

The genetic data were extracted from the blood draws taken during home visits. The genome-wide genotyping was performed at University College London Genomics in 2013–14 using the Illumina HumanOmni2.5 BeadChips (HumanOmni2.5-4v1, HumanOmni2.5-8v1.3), which measures approximately 2 million markers that capture the genomic variation down to 2.5% minor allele frequency (MAF).

#### Quality control

Single-nucleotide polymorphism (SNPs) were excluded if they were non-autosomal, MAF was <1%, if more than 2% of genotype data were missing, and if the Hardy–Weinberg Equilibrium *p* < 10^−4^. To single out the impact of *APOE*-ε4 and polygenic load separately, we excluded *APOE*, Lactase Gene, human leukocyte antigen, and 2 inversion regions located on 8p23.1 and 17q21.31 (40) (Supplementary Material). Furthermore, samples were removed based on call rate (<0.99), heterozygosity, and relatedness and if the recorded sex phenotype was inconsistent with genetic sex (Supplementary Table 3). To improve genome coverage, we imputed untyped quality-controlled genotypes to the Haplotype Reference Consortium (41,42) using the University of Michigan Imputation Server (41). Post-imputation, we kept variants that were genotyped or imputed at INFO > 0.80 leading to *n* = 7 179 780 variants being retained for further analyses. To investigate population structure, principal components analysis was conducted (43,44). We retained 10 principal components to account for any ancestry differences in genetic structures that could bias results (43,44).

#### Polygenic score

To calculate PGS_longevity_, we used summary statistics from the most recent GWAS of a longevity phenotype including 11 262 participants surviving at or beyond the age corresponding to the 90^th^ survival percentile, and 25 483 participants whose age at death or at last contact was at or below the age corresponding to the 60^th^ survival percentile (11). We calculated PGS_longevity_ using the thresholding method; here, PGSs are calculated as a weighted sum of the allele dosages, summing over the common markers abiding by the *P* value thresholds (*P*_Ts_) weighted according to the strength of effect estimate. To decide which *P*_T_ for PGS to take forward for further analyses, using information on sample size (*n*), total number of independent markers (*m*), and lower and upper *P*-values, we estimated the power encompassed in each *P*_T_ (Supplementary Table 4) (45). Our estimates showed that the ultimate *P*_T_ was 0.001 (*m* = 2 217, *R*^2^ = 0.135, *P* = 3.19 × 10^−77^), which was used in the subsequent analyses. To aid the interpretability of the results, PGS_longevity_ was centered by subtracting the mean and multiplied by its corresponding standard deviation; this scaling led to a unit increase doubling the likelihood of survival (46).

#### APOE-ε4 status

In accordance with previous research (47). *APOE*-ε4 status was defined according to absence (*APOE* ε2/2, ε2/3, and ε3/3) or presence (*APOE* ε2/4, ε3/4, and ε4/4) of ε4 alleles. There were no significant differences in sociodemographic characteristics between participants with and without *APOE*-ε4 (Supplementary Table 5). There was a small correlation between PGS_longevity_ and *APOE*-ε4 (−0.08, 95% CI: −0.10 to −0.06).

#### Covariates

The set of covariates included age, sex, and 10 principal components. We tested for an interaction effect between PGS_longevity_ and sex, and *APOE*-ε4 status and sex, none of which were significant. Therefore, we did not include these interactions in the models. We also tested age^2^ and age^3^, age × gender terms significance. They were not significant and did not improve the model fit, hence we concluded that linear terms were sufficient for the models.

### Statistical Analyses

#### Power calculations

To ensure our analyses were well-powered, we calculated a minimal impact size that could be detected in our sample with the power of 0.80 and type I error of 0.05 using powerSurvEpi R package (Supplementary Material and Supplementary Table 6) (48).

#### Survival analyses

In survival analysis, a competing risk is an event the occurrence of which precludes the occurrence of the primary event of interest (49). For example, if the primary outcome of interest is time to death due to cardiovascular causes, then death due to noncardiovascular causes is a competing risk. To investigate the relationship of PGS_longevity_ with specific causes of mortality in the presence of competing risks, we used the cause-specific Cox proportional hazards (PH) model (50) and subdistribution Fine and Gray model (51). Before we ran the Cox PH model, we checked that all assumptions for this model were met using the Schoenfeld residuals test (52), which they were (Supplementary Table 7). In cause-specific Cox PH model, the cause-specific hazard ratio denotes the relative change in the instantaneous rate of the occurrence of the primary event among those alive. The cause-specific hazard ratios can be estimated by Cox PH models, where the event of interest is treated as the outcome, while all other mortality causes are deemed to be censoring events (50). In contrast to the Cox PH model, the subdistribution Fine and Gray model focuses on cumulative distribution functions, or a probability of a cause-specific event by a certain time. The model estimates the subdistribution hazard ratios, which show the relative change in the instantaneous rate of the event of interest in those adults who are event-free or who have experienced a competing event (51); though, the model has been criticized for the tendency to overestimate the chances of failure (49,51). It is argued that to develop a greater understanding of the primary outcome and the competing events relationship, both cause-specific and subdistribution hazard models ought to be fitted (53); thus, we present results from Cox PH and the Fine–Gray models.

#### Mediation analysis

**Figure 1.**
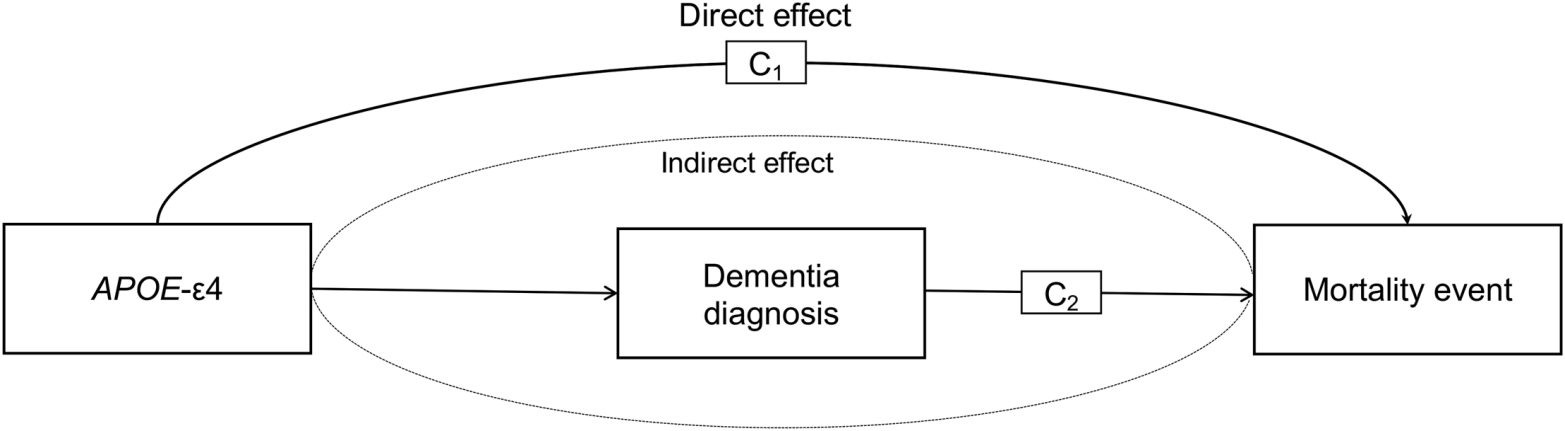
Diagram for mediation and confounding in the analysis of the impact of the *APOE*-ε4 on mortality event mediated by dementia diagnosis. This shows the paths between the exposure (ie, *APOE*-ε4), the mediator (ie, dementia diagnosis) and the outcome (ie, causes of mortality). C_1_ represents exposure-outcome confounders, and C_2_ represents mediator-outcome confounders. The direct effect encompasses the path between the exposure and the outcome; whereas indirect effect encompassed the path where the relationship between the exposure and the outcome is mediated by a mediator; here, an assumption of temporal ordering is met as dementia diagnosis was measured prior to the outcome. C_1_: the model was adjusted for *APOE*-ε4. C_2_: the model was adjusted for dementia diagnosis and all covariates.

To understand the pathways, whereby an exposure leads to an outcome, we conducted mediation analyses, repeated separately for each cause of mortality separately. We followed the most recent recommendations (54) and used a counterfactual mediation method implemented in the *regmedint* R package (55,56). In constant to the traditional mediation approaches (57), which were criticized for low statistical power, counterfactual methods accurately estimate direct and indirect effects irrespective of the statistical models and possible interactions (54,58). To be a mediator ***M***, a variable is likely to be a step in the chain of events, or pathways, between the exposure ***X*** and the outcome ***Y*** (59). In our analysis, ***X*** is *APOE*-ε4, ***M*** is dementia diagnosis, and ***Y*** is a mortality outcome (eg, all-cause mortality); here, an assumption of temporal ordering is met as dementia diagnosis was measured prior to a mortality event. Our assessment of mediation involved disentangling a direct effect and indirect effect (Figure 1) (60); the latter was used to ensure that the estimates provided are unbiased as it is arguably unrealistic to assume that the exposure and mediator do not interact in their effects on the outcome (54,58,60). In counterfactual analysis, a direct effect shows outcome change when exposure ***X*** moved from 0 to 1, while ***M*** is set at the level it would have been in the absence of ***X***; an indirect effect estimates outcome change if ***X*** is controlled at 1, but ***M*** changes from the value it would have been if ***X*** was 0, to the value at exposure level 1 (61). In the present study, the change in ***M*** was estimated using a logistic regression with the dementia diagnosis included as an outcome, and *APOE*-ε4 included in the model as an independent variable; whereas the change in ***Y*** was estimated with the Cox model whereas adjusted for PGS_longevity_, age, sex, and genetic ancestry. The mediation is present if both path ***a*** and path ***b*** as shown in Figure 1 are significant.

#### Sensitivity analyses

To limit the overriding influence of age in a “cohort of survivors,” we repeated all analyses as described earlier restricting the sample to the participants who were ≤75 years old at baseline. We further investigated if the findings observed in the main analyses were due to old age by repeating the analyses in the sample who were >75 years of age at baseline. The baseline sample characteristics of ELSA participants stratified by age are shown in Supplementary Table 8. We additionally investigated the gender influence on the relationships of all-cause mortality, and cause-specific mortality, with *APOE*-ε4 status, and polygenic predisposition to longevity as well as mediating effect of dementia diagnosis on these potential associations (the sample characteristics stratified by gender are shown in Supplementary Table 8). Finally, we investigated the effect of the dementia diagnosis on mortality using the Cox HP model adjusting the model sex, PC, and age. In terms of correction for multiple testing, it has been emphasized that adjustments for multiple testing are required in confirmatory studies whenever results from multiple tests have to be combined in one final conclusion (62). Because this study was not a confirmatory study, adjusting our results for multiple testing was not necessary; instead, we presented confidence intervals, which is in line with the recent guidelines for statistical reporting (62). All analyses were conducted in R version 4.1.2; all tests for analyses were 2-tailed; *p* ≤ .05 were considered statistically significant.

## Results

The total sample comprised 7 131 individuals for whom the quality-controlled genome-wide genotyping and data on all-cause mortality were available (Table 1). The baseline mean age for the entire sample was 64.7 years old (*SD* = 9.5, median = 63.0, IQR = 57.0–71.0, range 50–101); 46.2% (*n* = 3 292) were men. Of the entire sample, *n* = 1 234 (17.3%) died by the end of the 10-year follow-up period. The most common cause of death was due to cancers (34.8%) followed by CVD (29.7%) and respiratory diseases (15.9%) with the remaining mortalities 19.5% (*n* = 241) being attributed to all other causes. Of the whole sample, *n* = 194 (2.7%) adults were diagnosed with dementia by the end of the mean 10-year follow-up period (Supplementary Table 2). Of those older adults who were diagnosed with dementia, 45.4% (*n* = 88) were aged 75 years old and younger, and *n* = 106 (54.6%) were older than 75 years old at the time of diagnosis (Supplementary Table 9).

**Table 1.** Baseline Sample Characteristics of English Longitudinal Study of Aging (ELSA) Participants

Baseline characteristics	Total sample	Mortality event		Test statistics	
	*n* = 7 131	No	Yes		
		*n* = 5 897 (82.7%)	*n* = 1 234 (17.3%)		
	Mean (*SD*)/*n* (%)	Mean (*SD*)/*n* (%)	Mean (*SD*)/*n* (%)	*t*(*df*)/*x*^2^(*df*)	*p* Value
Length of follow-up, y	9.1 (2.0)	9.9 (0.3)	5.7 (2.7)	115.63 (7 129)	<.001
Age (y)	64.7 (9.5)	62.7 (8.2)	74.2 (9.5)	−43.49 (7 129)	<.001
Gender					
Men	3 292 (46.2)	2 618 (44.4)	674 (54.6)	42.92 (1)	<.001
Women	3 839 (53.8)	3 279 (55.6)	560 (45.4)		
Dementia diagnosis	194 (2.7%)	45 (0.8%)	149 (8.4%)	291.5 (1)	<.001
Cause of mortality event					
Any	—	—	1 234 (100.0)		
Cancer	—	—	430 (34.8)		
CVD	—	—	367 (29.7)		
Respiratory	—	—	196 (15.9)		
Other	—	—	241 (19.5)		

*Note*: CVD = cardiovascular disease; *df* = degrees of freedom; *SD* = standard deviation. Statistically significant at *p* <.05.

### PGS_longevity_, *APOE*-ε4, All-Cause Mortality, and Cause-Specific Mortality

In the Cox PH model, a 1-*SD* increase in PGS_longevity_ was associated with a reduced risk for all-cause mortality by an average of 7% (Hazard ratio [HR] = 0.93, 95% CI: 0.88–0.98, *p* = .010) during the 10-year follow-up (Table 2). In cause-specific analyses, 1-*SD* increase in PGS_longevity_ was associated with a lower hazard for mortality attributed to all other causes by an average of 19% (HR = 0.81, 95% CI: 0.71–0.93, *p* = .002) at the end of the 10-year follow-up period. Similar results were observed in the Fine–Gray model (Table 2). *APOE*-ε4 status was not associated with all-cause mortality, and cause-specific mortality, in the Cox PH and Fine–Gray models (Table 2).

**Table 2.** Survival Analyses Highlighting Associations Between PGS and *APOE*-ε4 and Risk for Cause-Specific Mortality During The Average 10-y Follow-up Period

	All cause	Cancer	CVD	Respiratory	Other
Cause-specific Cox model	HR^*^ (95% CI), *p* Value	HR^*^ (95% CI), *p* Value	HR^*^ (95% CI), *p* Value	HR^*^ (95% CI), *p* Value	HR^1^ (95% CI), *p* Value
PGS_longevity_	0.93 (0.88, 0.98), .010	0.95 (0.87, 1.05), .333	0.96 (0.87, 1.06), .447	0.94 (0.81, 1.08), .374	0.81 (0.71, 0.93), .002
*APOE*-ε4	1.00 (0.88, 1.14), .991	0.97 (0.78, 1.22), .818	1.01 (0.79, 1.28), .953	0.70 (0.48, 1.01), .053	1.30 (0.98, 1.72), .064

Fine–Gray model	HR^†^ (95% CI), *p* Value	HR^†^ (95% CI), *p* Value	HR^†^ (95% CI), *p* Value	HR^†^ (95% CI), *p* Value	HR^2^ (95% CI), *p* Value
PGS_longevity_	—	0.96 (0.87, 1.06), .400	0.98 (0.88, 1.08), .660	0.95 (0.82, 1.09), .450	0.81 (0.70, 0.94), .005
*APOE*-ε4	—	0.98 (0.78, 1.23), .870	1.03 (0.80, 1.31), .830	0.70 (0.48, 1.01), .057	1.31 (0.99, 1.74), .057

*Notes*: *APOE*-ε4 = ε4 allele of the apolipoprotein E gene; CI = confidence intervals; CVD = cardiovascular disease; HR = hazard ratio; PGS = polygenic score for longevity. The analyses are based on the entire sample. Statistically significant at *p* <.05.

^*^Cause-specific hazard ratios estimated by the cause-specific Cox models.

^†^Sub-distribution hazard ratios estimated by the Fine–Gray model.

### Mediation Analyses

**Figure 2.**
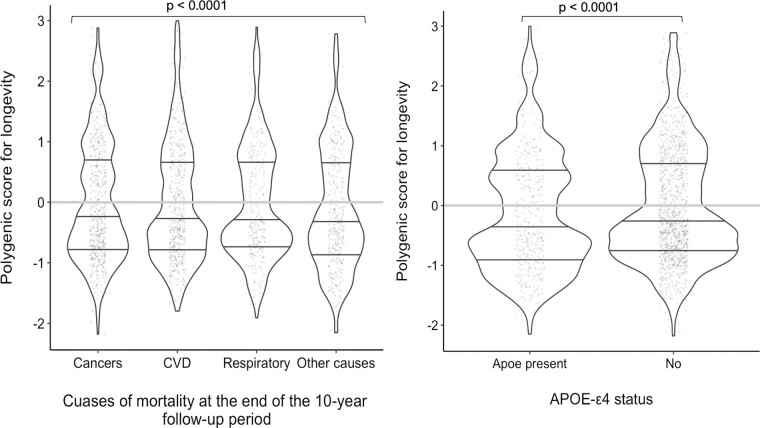
Distribution of the polygenic score for longevity by cause specific mortalities and by the presence or absence of *APOE*-ε4. This figure visualizes the distribution of polygenic score for longevity across cause-specific mortalities and *APOE*-ε4 status using Violin plot, which is a hybrid of a box plot and a kernel density plot. In this figure, each violin contains 3 horizontal lines, which represent lower quartile, middle quartile (median), and upper quartile, respectively; the horizontal gray line represents the mean. Wider sections of the violin plot represent a higher probability that members of the cohort will take on the given value; the skinnier sections represent a lower probability.

The distribution of PGS_longevity_ by cause-specific mortalities and by *APOE*-ε4 status is presented in Figure 2 and Supplementary Figure 2. Although there was no significant direct effect of *APOE*-ε4 status on mortality risk, nor each cause of mortality included in the analyses (Table 3), there was a significant chained mediation effect of *APOE*-ε4 status and causes of mortality related to all other causes than those due to cancers, CVD, respiratory diseases through dementia diagnosis (Indirect effect: HR = 1.03, 95% CI: 1.00, 1.06, *p* = .030) with a total percent mediated by dementia of 24.0% (Table 3).

**Table 3.** Mediation Analysis for the Impact of the *APOE*-ε4 on Specific Causes of Mortality in the Following the Average 10 y Mediated by a Dementia Diagnosis

Causes of mortality	All	Cancer	CVD	Respiratory	Other causes
	HR (95% CI), *p* Value	HR (95% CI), *p* Value	HR (95% CI), *p* Value	HR (95% CI), *p* Value	HR (95% CI), *p* Value
Direct effect	1.00 (0.99, 1.01), .662	1.00 (0.99, 1.01), .795	1.00 (0.998, 1.01), .665	1.004 (0.998.1.01), .064	1.03 (1.00, 1.06), .565
Indirect effect	1.00 (0.99, 1.01), .180	0.99 (0.99, 1.01), .526	0.99 (0.99, 1.00), .351	1.00 (0.99, 1.02), .724	1.03 (1.00, 1.06), .030
Total effect	0.97 (0.85, 1.12), .702	0.97 (0.77, 1.21), .775	1.05 (0.82, 1.35), .683	0.70 (0.48, 1.02), .063	1.13 (0.82, 1.56), .446
Percent mediated by dementia	−15.0%	8.0%	−7.0%	−1.0%	24.0%

*Notes*: *APOE*-ε4 = ε4 allele of the apolipoprotein E gene; CI = confidence intervals; CVD = cardiovascular disease; HR = hazard ratio; OR = odds ratio.

### Sensitivity Analyses

Restricting analyses to participants aged ≤75 years old yielded different results in the following ways. First, in the Cox PH model, there was no significant association between PGS_longevity_ and “other” causes of death in the following 10 years of follow-up (HR = 0.89, 95% CI: 0.72–1.09, *p* = .245; Supplementary Table 10). Second, the relationship of *APOE*-ε4 status with “other” causes of mortality became significant (HR = 1.66, 95% CI: 1.10–2.51, *p* = .015; Supplementary Table 10). The mediation effect of the dementia diagnosis on the association between *APOE*-ε4 status and “Other” causes of mortality increased to 34% (Supplementary Table 11). When the analyses were restricted to participants who were aged >75 years old at baseline (Supplementary Tables 12 and 13), the results mirrored the findings presented in the main analyses.

Furthermore, the results of the survival and mediation analyses stratified by gender are shown in Supplementary Tables 14 and 15. In the Cox PH model, *APOE*-ε4 status was associated with a reduced risk for all-cause mortality (HR = 0.91, 95% CI: 0.83–0.99, *p* = .030), cancers (HR = 0.85, 95% CI: 0.73–0.99, *p* = .039) and other causes of mortality (HR = 0.79, 95% CI: 0.65–0.96, *p* = .014) in women only in the following 10-year follow-up. The diagnosis of dementia mediates the effect of *APOE*-ε4 status on other causes of mortality in women with the percent mediated being 34%; though the *p* value was .061 (Supplementary Table 15). Moreover, a 1-*SD* increase in PGS_longevity_ was associated with a decreased risk for mortalities related to cancers in women by an average of 33% (HR = 0.67, 95% CI: 0.46–0.98, *p* = .035) during the 10-year follow-up; this finding was confirmed in Fine–Gray model. As deaths caused by dementia had been part of “other” mortality events, it was expected that the dementia diagnosis would be strongly associated with “other” causes of mortality; this was indeed the case (Supplementary Table 16).

## Discussion

To our knowledge, this is the first study to investigate the impact of polygenic predisposition to longevity and *APOE*-ε4 status on all-cause mortality, and specific causes of mortality, in a large population-representative sample of adults aged ≥50 years old during a 10-year follow-up period. To determine how an aggregate of common genetic markers of small effect and a rare genetic marker of large effect in the genome contribute to causes of mortality, we differentiated between PGS_longevity_ and *APOE*-ε4 from one another. Having used mediation analyses, we further assessed the relative magnitude of different pathways by which *APOE*-ε4 status, polygenic predisposition to longevity, and diagnosis of dementia influenced all-cause mortality, and specific causes of mortality, in older adults from the general population.

Consistent with the assertion that the genetic component of longevity is likely to be influenced by many common genetic markers (11,29), our results showed that one standard deviation increase in polygenic predisposition to longevity was associated with a decrease by an average of 7% in the risk for all-cause mortality in the following 10 years in the entire cohort. Although the observed polygenic contribution to all-cause mortality became nonsignificant in the sample limited to adults who were aged ≤75 years old, in the analyses encompassing adults aged > 75 years of age, our results showed that one standard deviation increase in polygenic predisposition to longevity was associated with a decrease by an average 24% in the risk for all-cause mortality in the following 10 years. These results imply that the contribution of multiple genetic markers, each with a weak to moderate input, to survival, is significant in adults who are older than 75 years of age. This, in turn, is consistent with previous studies showing that polygenic predisposition to longevity was associated with survival in adults who exceeded the average life expectancy, such as centenarians (11,28). This is arguably because those who carry the longevity-favoring genetic variants have a better chance of surviving to older ages (63); whereas, in those adults who have not reached such a survival age, other factors may be more important in influencing all-cause mortality risk (64). In cause-specific analyses, 1-*SD* increase in PGS_longevity_ was associated with a lower hazard for mortality attributed to all other causes by an average of 19% at the end of the 10-year follow-up period. Because the “other” causes of mortality category encompassed all other causes that were not related to cancers, respiratory diseases, and CVD, it is challenging to disentangle the true nature of this relationship with a polygenic predisposition to longevity. As it is likely that this category included deaths due to dementia (31,32), one of the potential explanations may be that higher PGS_longevity_ was associated with a lower risk of dying from dementia; though this would need to be further investigated in a larger sample.

Evidence from candidate gene studies and genome-wide association studies suggested that cardiovascular pathways were involved in longevity (65,66). However, in our cause-specific analyses, polygenic predisposition to longevity was not associated with mortalities that occurred due to CVD, nor respiratory-related diseases in the following 10 years. It was previously argued that common genetic markers for longevity acted additively in reducing cancer risk (67). In line with this, our results showed that a 1-*SD* increase in PGS_longevity_ was associated with a reduced risk for cancer-related mortalities by an average 33% in women but not in men in the following 10 years. Similar findings were obtained in previous genetic association studies of longevity where it was shown that results were not driven by the fact that, on average, women tend to live longer than men nor by the study sample size (63). There may be several explanations for this difference in polygenic influences on longevity in women and men. For example, some evidence suggests that men and women differ in their innate, humoral, and cell-mediated responses to viral challenges (68). As the sex differences in genetic influence on longevity are affected by different environmental factors and roles women and men lead in their life course (69), these factors ought also to be considered when interpreting the results.

Although *APOE*-ε4 was previously linked to cardiovascular diseases (including heart attack and stroke) (70), we did not observe a significant direct effect of *APOE*-ε4 on mortality due to cardiovascular illnesses. Similarly, *APOE*-ε4 was not associated with an increased risk of dying from respiratory-related disorders in adults with an average age of 64 years old. These results may imply that whereas *APOE*-ε4 is associated with getting a diagnosis for these health conditions, it does not influence the risk of dying from them. Nonetheless, our results showed that *APOE*-ε4 was associated with a decreased risk of dying due to cancers in women who were 50 years old and older. Even though the high expression of *APOE* was shown to promote tumor development, proliferation, and metastasis (71,72), previous studies demonstrated that in some cancers, such as ovarian cancer and melanoma, the high expression of *APOE* shows protective effects (73,74), which is consistent with our results. For example, *APOE* was shown to suppress metastasis by reducing the invasive behavior of cancer cells (75), inhibiting endothelial cell recruitment (74,75), and enhancing antitumor immunity by modulating myeloid immune cell populations (76). Furthermore, our results showed that *APOE*-ε4 was associated with a decreased risk for other causes of mortality in older men and women. Although some studies showed that *APOE*-ε4 was associated with having an elevated risk of mortality (77), in other population-based studies it was found that *APOE*-ε4 was not related to mortality risks (78,79). These differences in findings may be attributed to differences in environmental factors that were specific to the cohorts used in each study, which in turn may interact with *APOE* and act in a multifaceted way at different phases of life (80).

Mediation analyses further revealed that this relationship is mediated by dementia diagnosis. In fact, our analyses estimated that the percent excess risk of *APOE*-ε4 on other causes of mortality risk explained by the dementia diagnosis was 24%, which increased to 34% when we limited the sample to adults who were aged ≤75 years old and 32% when the analyses were restricted to women. These results are consistent with the accumulated evidence asserting that individuals who have dementia have excess mortality and a shorter life expectancy (81) compared to people without this diagnosis (82), especially among women in the United Kingdom (32). To ensure that these estimates were accurate, we controlled for all 3 main sources of potential bias that may cause mediation analyses to give flawed conclusions, such as mediator-outcome confounding, exposure–mediator interaction, and mediator-outcome confounding affected by the exposure (60). Consequently, because mediation assessment may help to identify different potential targets for early intervention, these findings are of clinical relevance. The findings of this study also produce quantitative estimates of this risk, allowing specific consideration of the potential impact on public health (83). Furthermore, work investigating more diverse populations will be necessary to support the extrapolation of these findings outside of the considered contexts.

Even though PGSs can be seen as unconfounded proxies for the lifetime predisposition to mortality, a gene-environmental correlation may still be present, which in turn may influence the mortality risk in the general population. The low generalizability of genetic studies across populations is noteworthy (84). This is because the construction of PGSs is mainly dependent on the availability of the summary statistics from GWASs, which are currently predominately based on European participants (84). Similarly, because PGSs are built on GWAS, they may be restricted by the same limiting factors that are inherent to GWASs, such as being unable to capture rare variants, poorly tagged or multiple independent variants, gene-by-gene interactions, and gene–environment correlation (85). Because of the relatively small number of dementia cases, we could not explore the types of dementia, as this may increase likelihood of false results due to multiple testing. To minimize chances of collider bias affecting our findings (86), all covariates that were included in the models were set at birth; on the other hand, however, we did not adjust the confounding effect of some other factors, such as smoking and educational attainment on the mortality risk. Nonetheless, the potentially mediating effects of these factors on the nexus of PGS_longevity_ and mortality could be assessed in future studies. Furthermore, *FOXO*3 is another candidate gene for longevity and part of the well-characterized the insulin/insulin-like growth factor signalling (IIS) pathway (87). However, in the present study, we focused on *APOE*-ε4 only and this gene has widespread effects on aging phenotypes, particularly cardiovascular disease, and dementia, and as such influences the ability to achieve a long and healthy life, making it a more appropriate candidate for analyses entailing these phenotypes. Finally, the reported associations may be influenced by the other correlated traits, which may be independently associated with all-cause mortality.

## Conclusion

Although polygenic predisposition to longevity was associated with all-cause mortality in the following 10 years in older adults, it was not related to mortality that occurred due to cardiovascular diseases, and respiratory-related disorders. Our analyses further support that the effect of *APOE*-ε4 on the risk for other causes of mortality is mediated by approximately up to one-third through dementia diagnosis. Our results contribute to a better understanding of underlying genetic mechanisms influencing all-cause mortality and specific causes of death in the general population of older adults. They further demonstrate that to reduce the mortality rate in adults who are aged 50 years old and older, it is essential to prevent dementia onset in the general population.

## Supplementary Material

glad168_suppl_Supplementary_Material

## Data Availability

The English Longitudinal Study of Aging (ELSA) was developed by a team of researchers based at University College London, the Institute for Fiscal Studies, and the National Centre for Social Research. The data sets generated and/or analyzed during the current study are available in UK Data Services and can be accessed at: https://discover.ukdataservice.ac.uk. No administrative permissions were required to access these data.

## Disclaimer

The views expressed in this publication are those of the authors and not necessarily those of the NHS, the National Institute for Health Research, or the Department of Health and Social Care. The NHS, the National Institute for Health Research, or the Department of Health and Social Care had no role in the design and conduct of the study; collection, management, analysis, and interpretation of the data; preparation, review, or approval of the manuscript; and the decision to submit the manuscript for publication.

## References

[1] Mathers CD , Boerma T, Ma Fat D. Global and regional causes of death. Br Med Bull. 2009;92:7–32. doi:10.1093/bmb/ldp028 19776034

[2] Reiner AP , Carlson CS, Jenny NS, et al. USF1 gene variants, cardiovascular risk, and mortality in European Americans: analysis of two US cohort studies. Arterioscler Thromb Vasc Biol. 2007;27(12):2736–2742. doi:10.1161/ATVBAHA.107.154559 17885212

[3] Reiner AP , Diehr P, Browner WS, et al. Common promoter polymorphisms of inflammation and thrombosis genes and longevity in older adults: the Cardiovascular Health Study. Atherosclerosis. 2005;181(1):175–183. doi:10.1016/j.atherosclerosis.2005.01.028 15939070

[4] Statistics, O.f.N. *Dementia and Alzheimer’s disease deaths including comorbidities, England and Wales: 2019 registrations*. 2020. https://www.ons.gov.uk/peoplepopulationandcommunity/birthsdeathsandmarriages/deaths/bulletins/dementiaandalzheimersdiseasedeathsincludingcomorbiditiesenglandandwales/2019registrations

[5] Guttmacher AE , Porteous ME, McInerney JD. Educating health-care professionals about genetics and genomics. Nat Rev Genet. 2007;8(2):151–157. doi:10.1038/nrg2007 17230201

[6] Tan Q , Jacobsen R, Sørensen M, Christiansen L, Kruse TA, Christensen K. Analyzing age-specific genetic effects on human extreme age survival in cohort-based longitudinal studies. Eur J Hum Genet. 2013;21(4):451–454. doi:10.1038/ejhg.2012.182 22892531 PMC3598313

[7] Hjelmborg vBJ , Iachine I, Skytthe A, et al. Genetic influence on human lifespan and longevity. Hum Genet. 2006;119(3):312–321. doi:10.1007/s00439-006-0144-y 16463022

[8] Wray NR , Lee SH, Mehta D, Vinkhuyzen AAE, Dudbridge F, Middeldorp CM. Research review: polygenic methods and their application to psychiatric traits. J Child Psychol Psychiatry. 2014;55(10):1068–1087. doi:10.1111/jcpp.12295 25132410

[9] Purcell SM , Wray NR, Stone JL, et al.; International Schizophrenia Consortium. Common polygenic variation contributes to risk of schizophrenia and bipolar disorder. Nature. 2009;460(7256):748–752. doi:10.1038/nature08185 19571811 PMC3912837

[10] Dudbridge F. Power and predictive accuracy of polygenic risk scores. PLoS Genet. 2013;9(3):e1003348. doi:10.1371/journal.pgen.1003348 23555274 PMC3605113

[11] Deelen J , Evans DS, Arking DE, et al. A meta-analysis of genome-wide association studies identifies multiple longevity genes. Nat Commun. 2019;10(1):3669. doi:10.1038/s41467-019-11558-2 31413261 PMC6694136

[12] Mahley RW. Apolipoprotein E: cholesterol transport protein with expanding role in cell biology. Science. 1988;240(4852):622–630. doi:10.1126/science.3283935 3283935

[13] Nebel A , Kleindorp R, Caliebe A, et al. A genome-wide association study confirms *APOE* as the major gene influencing survival in long-lived individuals. Mech Ageing Dev. 2011;132(6–7):324–330. doi:10.1016/j.mad.2011.06.008 21740922

[14] Deelen J , Beekman M, Uh H-W, et al. Genome-wide association study identifies a single major locus contributing to survival into old age; the *APOE* locus revisited. Aging Cell. 2011;10(4):686–698. doi:10.1111/j.1474-9726.2011.00705.x 21418511 PMC3193372

[15] Broer L , Buchman AS, Deelen J, et al. GWAS of longevity in CHARGE consortium confirms *APOE* and *FOXO3* candidacy. J Gerontol A Biol Sci Med Sci. 2015;70(1):110–118. doi:10.1093/gerona/glu166 25199915 PMC4296168

[16] Ewbank DC. The *APOE* gene and differences in life expectancy in Europe. J Gerontol A Biol Sci Med Sci. 2004;59(1):16–20. doi:10.1093/gerona/59.1.b16 14718482

[17] Gerdes LU , Gerdes C, Hansen PS, Klausen IC, Faergeman O, Dyerberg J. The apolipoprotein E polymorphism in Greenland Inuit in its global perspective. Hum Genet. 1996;98(5):546–550. doi:10.1007/s004390050257 8882873

[18] Corbo RM , Scacchi R. Apolipoprotein E (*APOE*) allele distribution in the world. Is *APOE**4 a “thrifty” allele? Ann Hum Genet. 1999;63(Pt 4):301–310. doi:10.1046/j.1469-1809.1999.6340301.x 10738542

[19] Gomez-Coronado D , Alvarez JJ, Entrala A, Olmos JM, Herrera E, Lasunción MA. Apolipoprotein E polymorphism in men and women from a Spanish population: allele frequencies and influence on plasma lipids and apolipoproteins. Atherosclerosis. 1999;147(1):167–176. doi:10.1016/s0021-9150(99)00168-9 10525138

[20] Panza F , D'Introno A, Colacicco AM, et al. Vascular genetic factors and human longevity. Mech Ageing Dev. 2004;125(3):169–178. doi:10.1016/j.mad.2003.12.005 15013661

[21] Mahley RW , Rall SC Jr. Apolipoprotein E: far more than a lipid transport protein. Annu Rev Genomics Hum Genet. 2000;1:507–537. doi:10.1146/annurev.genom.1.1.507 11701639

[22] Wilson PW , Myers RH, Larson MG, Ordovas JM, Wolf PA, Schaefer EJ. Apolipoprotein E alleles, dyslipidemia, and coronary heart disease. The Framingham Offspring Study. JAMA. 1994;272(21):1666–1671. doi:10.1001/jama.1994.03520210050031 7966894

[23] Smith JD. Apolipoprotein E4: an allele associated with many diseases. Ann Med. 2000;32(2):118–127. doi:10.3109/07853890009011761 10766403

[24] Sando SB , Melquist S, Cannon A, et al. *APOE* epsilon 4 lowers age at onset and is a high risk factor for Alzheimer’s disease; a case control study from central Norway. BMC Neurol. 2008;8:9. doi:10.1186/1471-2377-8-9 18416843 PMC2375917

[25] Corder EH , Saunders AM, Strittmatter WJ, et al. Gene dose of apolipoprotein E type 4 allele and the risk of Alzheimer’s disease in late onset families. Science. 1993;261(5123):921–923. doi:10.1126/science.8346443 8346443

[26] Helkala EL , Koivisto K, Hanninen T, et al. Memory functions in human subjects with different apolipoprotein E phenotypes during a 3-year population-based follow-up study. Neurosci Lett. 1996;204(3):177–180. doi:10.1016/0304-3940(96)12348-x 8938259

[27] Torkamani A , Wineinger NE, Topol EJ. The personal and clinical utility of polygenic risk scores. Nat Rev Genet. 2018;19(9):581–590. doi:10.1038/s41576-018-0018-x 29789686

[28] Tesi N , van der Lee SJ, Hulsman M, et al. Polygenic risk score of longevity predicts longer survival across an age continuum. J Gerontol A Biol Sci Med Sci. 2021;76(5):750–759. doi:10.1093/gerona/glaa289 33216869 PMC8087277

[29] Kirkwood TB , Cordell HJ, Finch CE. Speed-bumps ahead for the genetics of later-life diseases. Trends Genet. 2011;27(10):387–388. doi:10.1016/j.tig.2011.07.001 21824675

[30] Steptoe A , Breeze E, Banks J, Nazroo J. Cohort profile: the English Longitudinal Study of Ageing. Int J Epidemiol. 2013;42(6):1640–1648. doi:10.1093/ije/dys168 23143611 PMC3900867

[31] Dewey ME , Saz P. Dementia, cognitive impairment and mortality in persons aged 65 and over living in the community: a systematic review of the literature. Int J Geriatr Psychiatry. 2001;16(8):751–761. doi:10.1002/gps.397 11536341

[32] Office for National Statistics, Deaths Registered in England and Wales (Series DR). 2014.

[33] Cadar D , Lassale C, Davies H, Llewellyn DJ, Batty GD, Steptoe A. Individual and area-based socioeconomic factors associated with dementia incidence in England: evidence from a 12-year follow-up in the English Longitudinal Study of Ageing. JAMA Psychiatry. 2018;75:723. doi:10.1001/jamapsychiatry.2018.1012 29799983 PMC6145673

[34] Jorm AF. A short form of the Informant Questionnaire on Cognitive Decline in the Elderly (IQCODE): development and cross-validation. Psychol Med. 1994;24(1):145–153. doi:10.1017/s003329170002691x 8208879

[35] Quinn TJ , Fearon P, Noel-Storr AH, Young C, McShane R, Stott DJ. Informant Questionnaire on Cognitive Decline in the Elderly (IQCODE) for the diagnosis of dementia within community dwelling populations. Cochrane Database Syst Rev. 2014(4):CD010079. doi:10.1002/14651858.CD010079.pub2 24719028

[36] Deckers K , Cadar D, van Boxtel MPJ, Verhey FRJ, Steptoe A, Köhler S. Modifiable risk factors explain socioeconomic inequalities in dementia risk: evidence from a population-based prospective cohort study. J Alzheimer’s Dis. 2019;71:549–557. doi:10.3233/jad-190541 31424404 PMC6839472

[37] Hackett RA , Steptoe A, Cadar D, Fancourt D. Social engagement before and after dementia diagnosis in the English Longitudinal Study of Ageing. PLoS One. 2019;14(8):e0220195. doi:10.1371/journal.pone.0220195 31369590 PMC6675105

[38] Rafnsson SB , Orrell M, d’Orsi E, Hogervorst E, Steptoe A. Loneliness, social integration, and incident dementia over 6 years: prospective findings from the English Longitudinal Study of Ageing. J Gerontol B Psychol Sci Soc Sci. 2017;75:114–124. doi:10.1093/geronb/gbx087 PMC6909434 28658937

[39] Ajnakina O , Cadar D, Steptoe A. Interplay between socioeconomic markers and polygenic predisposition on timing of dementia diagnosis. J Am Geriatr Soc. 2020;68:1529–1536. doi:10.1111/jgs.16406 32187654 PMC7363562

[40] Novembre J , Johnson T, Bryc K, et al. Genes mirror geography within Europe. Nature. 2008;456(7218):98–101. doi:10.1038/nature07331 18758442 PMC2735096

[41] Das S , Forer L, Schönherr S, et al. Next-generation genotype imputation service and methods. Nat Genet. 2016;48(10):1284–1287. doi:10.1038/ng.3656 27571263 PMC5157836

[42] McCarthy S , Das S, Kretzschmar W, et al.; Haplotype Reference Consortium. A reference panel of 64,976 haplotypes for genotype imputation. Nat Genet. 2016;48(10):1279–1283. doi:10.1038/ng.3643 27548312 PMC5388176

[43] Price AL , Patterson NJ, Plenge RM, Weinblatt ME, Shadick NA, Reich D. Principal components analysis corrects for stratification in genome-wide association studies. Nat Genet. 2006;38(8):904–909. doi:10.1038/ng1847 16862161

[44] Wang D , Sun Y, Stang P, Berlin JA, Wilcox MA, Li Q. Comparison of methods for correcting population stratification in a genome-wide association study of rheumatoid arthritis: principal-component analysis versus multidimensional scaling. BMC Proc. 2009;3(Suppl 7):S109. doi:10.1186/1753-6561-3-s7-s109 20017973 PMC2795880

[45] Palla L , Dudbridge F. A fast method that uses polygenic scores to estimate the variance explained by genome-wide marker panels and the proportion of variants affecting a trait. Am J Hum Genet. 2015;97(2):250–259. doi:10.1016/j.ajhg.2015.06.005 26189816 PMC4573448

[46] Reginsson GW , Ingason A, Euesden J, et al. Polygenic risk scores for schizophrenia and bipolar disorder associate with addiction. Addict Biol. 2018;23(1):485–492. doi:10.1111/adb.12496 28231610 PMC5811785

[47] Zhang A , Zhao Q, Xu D, Jiang S. Brain *APOE* expression quantitative trait loci-based association study identified one susceptibility locus for Alzheimer’s disease by interacting with *APOE* epsilon4. Sci Rep. 2018;8(1):8068. doi:10.1038/s41598-018-26398-1 29795290 PMC5966425

[48] Qiu W , et al. powerSurvEpi: power and sample size calculation for survival analysis of epidemiological studies. R package version 0.1.3. 2021.

[49] Austin PC , Fine JP. Practical recommendations for reporting Fine-Gray model analyses for competing risk data. Stat Med. 2017;36(27):4391–4400. doi:10.1002/sim.7501 28913837 PMC5698744

[50] Cox DR. Regression models and life-tables. J R Statist Soc B (Methodol.). 1972;34(2):187–202. doi:10.1111/j.2517-6161.1972.tb00899.x

[51] Austin PC , Steyerberg EW, Putter H. Fine-Gray subdistribution hazard models to simultaneously estimate the absolute risk of different event types: cumulative total failure probability may exceed 1. Stat Med. 2021;40(19):4200–4212. doi:10.1002/sim.9023 33969508 PMC8360146

[52] Therneau GT. Proportional hazards tests and diagnostics based on weighted residuals. Biometrika. 1994;81:515–526. doi:10.1093/biomet/81.3.515

[53] Latouche A , Allignol A, Beyersmann J, Labopin M, Fine JP. A competing risks analysis should report results on all cause-specific hazards and cumulative incidence functions. J Clin Epidemiol. 2013;66(6):648–653. doi:10.1016/j.jclinepi.2012.09.017 23415868

[54] Vo TT , Superchi C, Boutron I, Vansteelandt S. The conduct and reporting of mediation analysis in recently published randomized controlled trials: results from a methodological systematic review. J Clin Epidemiol. 2020;117:78–88. doi:10.1016/j.jclinepi.2019.10.001 31593798

[55] Li Y , Mathur MB, Yoshida K. R package regmedint: extension of regression-based causal mediation analysis with effect measure modification by covariates. OFS Prints. 2022:1–27. doi:10.31219/osf.io/d4brv

[56] Yoshida K , Mathur M, Glynn RJ. Conducting regression-based causal mediation analysis using the R package“ regmedint”. OSF Prints. 2020:1–5. doi:10.31219/osf.io/6c79f

[57] Baron RM , Kenny DA. The moderator-mediator variable distinction in social psychological research: conceptual, strategic, and statistical considerations. J Pers Soc Psychol. 1986;51(6):1173–1182. doi:10.1037//0022-3514.51.6.1173 3806354

[58] Zhao X , Lynch JG Jr, Chen Q. Reconsidering Baron and Kenny: myths and truths about mediation analysis. J Cons Res. 2010;37(2):197–206. doi:10.1086/651257

[59] Greenland S , Neutra R. Control of confounding in the assessment of medical technology. Int J Epidemiol. 1980;9(4):361–367. doi:10.1093/ije/9.4.361 7203778

[60] Richiardi L , Bellocco R, Zugna D. Mediation analysis in epidemiology: methods, interpretation and bias. Int J Epidemiol. 2013;42(5):1511–1519. doi:10.1093/ije/dyt127 24019424

[61] Valeri L , Vanderweele TJ. Mediation analysis allowing for exposure-mediator interactions and causal interpretation: theoretical assumptions and implementation with SAS and SPSS macros. Psychol Methods. 2013;18(2):137–150. doi:10.1037/a0031034 23379553 PMC3659198

[62] Bender R , Lange S. Adjusting for multiple testing--when and how? J Clin Epidemiol. 2001;54(4):343–349. doi:10.1016/s0895-4356(00)00314-0 11297884

[63] Zeng Y , Nie C, Min J, et al. Sex differences in genetic associations with longevity. JAMA Netw Open. 2018;1(4):e181670. doi:10.1001/jamanetworkopen.2018.1670 30294719 PMC6173523

[64] Cooper R , Strand BH, Hardy R, Patel KV, Kuh D. Physical capability in mid-life and survival over 13 years of follow-up: British birth cohort study. BMJ. 2014;348:g2219. doi:10.1136/bmj.g2219 24787359 PMC4004787

[65] Pilling LC , Atkins JL, Bowman K, et al. Human longevity is influenced by many genetic variants: evidence from 75,000 UK Biobank participants. Aging. 2016;8(3):547–560. doi:10.18632/aging.100930 27015805 PMC4833145

[66] Revelas M , Thalamuthu A, Oldmeadow C, et al. Review and meta-analysis of genetic polymorphisms associated with exceptional human longevity. Mech Ageing Dev. 2018;175:24–34. doi:10.1016/j.mad.2018.06.002 29890178

[67] Yashin AI , Wu D, Arbeev KG, Ukraintseva SV. Polygenic effects of common single-nucleotide polymorphisms on life span: when association meets causality. Rejuvenation Res. 2012;15(4):381–394. doi:10.1089/rej.2011.1257 22533364 PMC3419841

[68] Klein SL , Jedlicka A, Pekosz A. The Xs and Y of immune responses to viral vaccines. Lancet Infect Dis. 2010;10(5):338–349. doi:10.1016/S1473-3099(10)70049-9 20417416 PMC6467501

[69] Harris KM , Halpern CT, Hussey J, et al. Social, behavioral, and genetic linkages from adolescence into adulthood. Am J Public Health. 2013;103(Suppl 1):S25–S32. doi:10.2105/AJPH.2012.301181 23927505 PMC3786750

[70] Lahoz C , Schaefer EJ, Cupples LA, et al. Apolipoprotein E genotype and cardiovascular disease in the Framingham Heart Study. Atherosclerosis. 2001;154(3):529–537. doi:10.1016/s0021-9150(00)00570-0 11257253

[71] Kopylov AT , Stepanov AA, Malsagova KA, et al. Revelation of proteomic indicators for colorectal cancer in initial stages of development. Molecules. 2020;25(3):619. doi:10.3390/molecules25030619 32023884 PMC7036866

[72] Kemp SB , Carpenter ES, Steele NG, et al. Apolipoprotein E promotes immune suppression in pancreatic cancer through NF-kappaB-mediated production of CXCL1. Cancer Res. 2021;81(16):4305–4318. doi:10.1158/0008-5472.CAN-20-3929 34049975 PMC8445065

[73] Ostendorf BN , Bilanovic J, Adaku N, et al. Common germline variants of the human *APOE* gene modulate melanoma progression and survival. Nat Med. 2020;26(7):1048–1053. doi:10.1038/s41591-020-0879-3 32451497 PMC8058866

[74] Lai H , Zhao X, Qin Y, et al. FAK-ERK activation in cell/matrix adhesion induced by the loss of apolipoprotein E stimulates the malignant progression of ovarian cancer. J Exp Clin Cancer Res. 2018;37(1):32. doi:10.1186/s13046-018-0696-4 29458390 PMC5819228

[75] Pencheva N , Tran H, Buss C, et al. Convergent multi-miRNA targeting of *APOE* drives LRP1/LRP8-dependent melanoma metastasis and angiogenesis. Cell. 2012;151(5):1068–1082. doi:10.1016/j.cell.2012.10.028 23142051 PMC3753115

[76] Tavazoie MF , Pollack I, Tanqueco R, et al. LXR/*APOE* activation restricts innate immune suppression in cancer. Cell. 2018;172(4):825–840.e18. doi:10.1016/j.cell.2017.12.026 29336888 PMC5846344

[77] Ewbank DC. Mortality differences by *APOE* genotype estimated from demographic synthesis. Genet Epidemiol. 2002;22(2):146–155. doi:10.1002/gepi.0164 11788960

[78] Slooter AJ , Cruts M, Van Broeckhoven C, Hofman A, van Duijin CM. Apolipoprotein E and longevity: the Rotterdam Study. J Am Geriatr Soc. 2001;49(9):1258–1259. doi:10.1046/j.1532-5415.2001.49251.x 11559391

[79] Koivisto AM , Lempiäinen P, Koivisto K, et al. Apolipoprotein E phenotype alone does not influence survival in Alzheimer’s disease: a population-based longitudinal study. Neuroepidemiology. 2000;19(6):327–332. doi:10.1159/000026272 11060507

[80] Rosvall L , Rizzuto D, Wang H-X, Winblad B, Graff C, Fratiglioni L. *APOE*-related mortality: effect of dementia, cardiovascular disease and gender. Neurobiol Aging. 2009;30(10):1545–1551. doi:10.1016/j.neurobiolaging.2007.12.003 18237822

[81] Hill GB , Forbes WF, Lindsay J. Life expectancy and dementia in Canada: the Canadian Study of Health and Aging. Chronic Dis Can. 1997;18(4):166–167. 9445364

[82] Brayne C , Gao L, Dewey M, Matthews FE; Medical Research Council Cognitive Function and Ageing Study Investigators. Dementia before death in ageing societies--the promise of prevention and the reality. PLoS Med. 2006;3(10):e397. doi:10.1371/journal.pmed.0030397 17076551 PMC1626550

[83] Hafeman DM , Schwartz S. Opening the Black Box: a motivation for the assessment of mediation. Int J Epidemiol. 2009;38(3):838–845. doi:10.1093/ije/dyn372 19261660

[84] Martin AR , Kanai M, Kamatani Y, Okada Y, Neale BM, Daly MJ. Clinical use of current polygenic risk scores may exacerbate health disparities. Nat Genet. 2019;51(4):584–591. doi:10.1038/s41588-019-0379-x 30926966 PMC6563838

[85] Reynolds CA , Finkel D. A meta-analysis of heritability of cognitive aging: minding the “missing heritability” gap. Neuropsychol Rev. 2015;25(1):97–112. doi:10.1007/s11065-015-9280-2 25732892 PMC5289922

[86] Arnold KF , Davies V, de Kamps M, Tennant PWG, Mbotwa J, Gilthorpe MS. Reflection on modern methods: generalized linear models for prognosis and intervention-theory, practice and implications for machine learning. Int J Epidemiol. 2021;49(6):2074–2082. doi:10.1093/ije/dyaa049 32380551 PMC7825942

[87] Anselmi CV , Malovini A, Roncarati R, et al. Association of the FOXO3A locus with extreme longevity in a southern Italian centenarian study. Rejuvenation Res. 2009;12(2):95–104. doi:10.1089/rej.2008.0827 19415983

