# Supplementary material for: Polygenic Propensity for Longevity, APOE-ε4 Status, Dementia Diagnosis, and Risk for Cause-Specific Mortality: A Large Population-Based Longitudinal Study of Older Adults
Source: J Gerontol A Biol Sci Med Sci. 2023 Jul 12;78(11):1973–82. doi: 10.1093/gerona/glad168 (PMC10613005; doi:10.1093/gerona/glad168)
Supplement: glad168_suppl_Supplementary_Material [file glad168_suppl_supplementary_material.docx]

**TABLE OF CONTENT**

| Power calculations | Page: 2 |
| --- | --- |
| GWAS QC based on SNP level | Page: 2 |
| **eFigure 1**. Cumulative rate of morality at each year of follow-up in ELSA | Page: 4 |
| **eFigure 2**. Distribution of polygenic score for longevity by cause specific mortalities in the presence or absence of APOE-ε4 | Page: 5 |
| **eTable 1**. The rate of mortality at each year of follow-up | Page: 6 |
| **eTable 2**. Comparisons between the ELSA participants who were included in the analyses and those who were excluded | Page: 7 |
| **eTable 3**. An overview of the summary of full QC procedure employed in the ELSA study and how many variants and/or participants were lost at each step | Page: 8 |
| **eTable 4**. Estimated the predictive accuracy (*R*^2^, p-value) for polygenic score for longevity | Page: 9 |
| **eTable 5**. Baseline sample characteristics of ELSA participants stratified by *APOE*-ε4 status | Page: 10 |
| **eTable 6**. Minimal Impact Size which can be found as statistically significant in the analytical sample with the power of 0.80 and type I error of 0.05 | Page: 11 |
| **eTable 7**. The statistics to show that assumptions for cox model were met | Page: 12 |
| **eTable 8**. Baseline sample characteristics of ELSA participants stratified by age | Page: 13 |
| **eTable 9**. Comparisons of the ELSA participants by gender. | Page: 14 |
| **eTable 10**. Cox regression analyses highlighting associations between PGS_longevity_ and *APOE*-ε4 and risk for cause-specific mortality during the 10-year follow-up period; the analyses are limited to adults who are aged 75 years old and younger | Page: 15 |
| **eTable 11**. Mediation analysis for the impact of the *APOE*-ε4 on specific causes of mortality in the following 10 years mediated by a dementia diagnosis; the analyses were restricted to the participants who were aged 75 years old or younger at baseline. | Page: 16 |
| **eTable 12**. Cox regression analyses highlighting associations between PGS_longevity_ and *APOE*-ε4 and risk for cause-specific mortality during the 10-year follow-up period; the analyses are limited to adults who were aged 76 years old or older at baseline. | Page: 17 |
| **eTable 13**. Mediation analysis for the impact of the *APOE*-ε4 on specific causes of mortality in the following 10 years mediated by a dementia diagnosis; the analyses were restricted to the participants who were aged 76 years old or older at baseline. | Page: 18 |
| **eTable 14.** Cox regression analyses highlighting associations between PGS_longevity_ and *APOE*-ε4 and risk for cause-specific mortality during the 10-year follow-up period stratified by gender. | Page: 19 |
| **eTable 15**. Mediation analysis for the impact of the *APOE*-ε4 on specific causes of mortality in the following 10 years mediated by a dementia diagnosis stratified by gender. | Page: 20 |
| **eTable 16.** Cox regression analyses highlighting associations between dementia diagnosis and risk for cause-specific mortality during the 10-year follow-up period. | Page: 21 |

*Power calculations.* To ensure our analyses were well-powered, we calculated a minimal impact size that could be detected in our sample with the power of 0.80 and type I error of 0.05 using powerSurvEpi R package.1 Minimal hazard ratios for APOE-ε4 for all-causes mortality was 1.20, 1.16 for cancers-related mortalities, 1.40 for CVD related mortalities, 1.67 for respiratory diseases related mortalities, 1.53 for other causes of mortality (Supplementary Table 6). A threshold for the protective hazard ratio for one standard deviation (1-SD) in PGS_longevity_ was 0.93 for all-cause mortality, 0.88 for cancers-related mortalities, 0.86 for CVD related mortalities, 0.82 for respiratory diseases related mortalities, and 0.83 for all other causes of mortality (Supplementary Table 6).

QC based on SNP level. Heterozygosity refers to carrying of two different alleles of a specific SNP. Excessive heterozygosity may imply a sample contamination, while less heterozygosity than expected may imply inbreeding2. In the ELSA study, the checks for heterozygosity were performed on a set of SNPs which were non-(highly) correlated. To generate a list of non-(highly) correlated SNPs, we excluded four regions that are known to contain clusters of highly correlated SNPs. These were the apolipoprotein E (APOE) gene (chromosome 19, 45,384,477 to 45,432,606 bp), Lactase Gene (LCT) (chromosome 6, 12578740 to 135837195 bp), human leukocyte antigen (HLA) (chromosome 2, 2550000 to 3350000 bp) and two inversion regions located on 8p23.1 (chromosome 8, 81305000 to 1200000 bp) and 17q21.31 (chromosome 17, 40900000-45000000 bp)3. We then pruned the SNPs using the ‘10 5 0.1’ parameters. These pruning parameters use a sliding window method that considers blocks of 10 SNPs and removes SNPs with r2 >0.10 afterward shifting the window by 5 SNPs. Those individuals with extremely low or high heterozygosity score (>3 standard deviations from the mean) were removed. Further, the genotyped data with a call rate of <98% was removed. SNPs in sex chromosomes and SNPs with a minor allele frequency (MAF) of <1% were excluded. SNPs whose genotype distributions deviated significantly from the Hardy-Weinberg equilibrium (HWE) (p<10-4) and with missingness <2% were also removed.

*References:*

1 Qiu, W., Chavarro, J., Lazarus, R., Rosner, B. & Ma, J. powerSurvEpi: Power and Sample Size Calculation for Survival Analysis of Epidemiological Studies. R package version 0.1.3. (2021).

2 Marees, A. T. *et al.* A tutorial on conducting genome-wide association studies: Quality control and statistical analysis. *Int J Methods Psychiatr Res* **27**, e1608, doi:10.1002/mpr.1608 (2018).

3 Novembre, J. *et al.* Genes mirror geography within Europe. *Nature* **456**, 98-101, doi:10.1038/nature07331 (2008).

**eFigure 1.** Cumulative rate of morality at each year of follow-up in ELSA

**
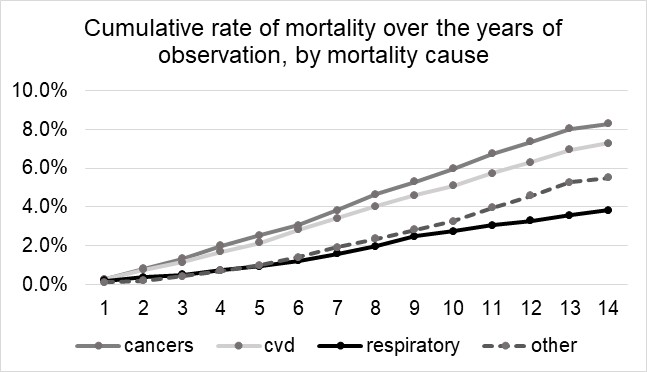
**

**eFigure 2.** Distribution of polygenic score for longevity by cause specific mortalities in the presence or absence of APOE-ε4

**
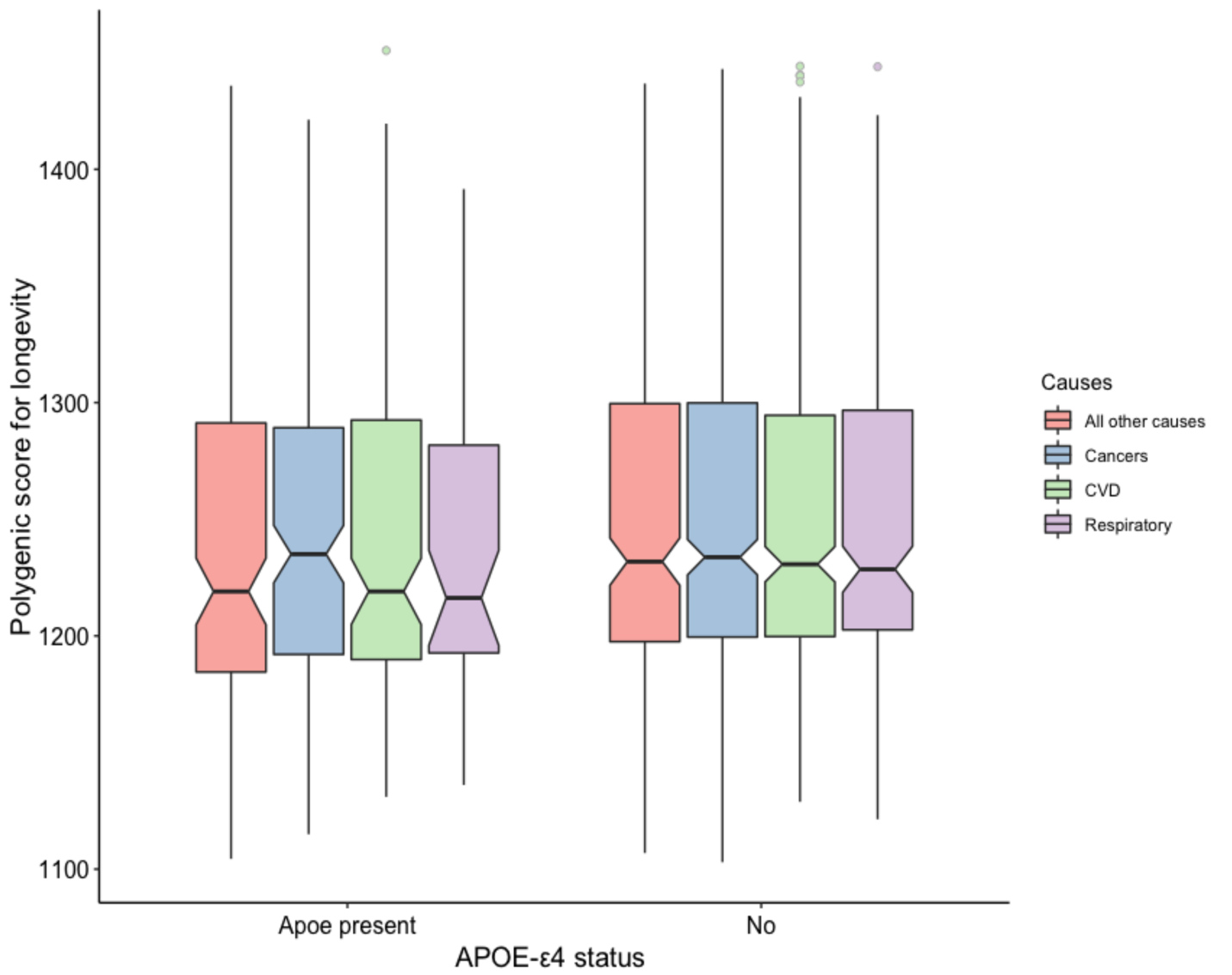
**

**eTable 1.** The rate of mortality at each year of follow-up

| **Year of participation in the study** | **Mortality** | | **Cause specific mortality** | | | |
| --- | --- | --- | --- | --- | --- | --- |
|  | **n (%) All causes** | **Age, Median (IQR)** | **n (%) Cancer** | **n (%) CVD** | **n (%) Respiratory** | **n (%) Other** |
| Year 1 | 61 (0.9) | 74.0 (14.0) | 20 (0.3) | 19 (0.3) | 14 (0.2) | 8 (0.1) |
| Year 2 | 94 (1.3) | 77.0 (12.5) | 38 (0.5) | 36 (0.5) | 13 (0.2) | 7 (0.1) |
| Year 3 | 90 (1.3) | 77.5 (15.0) | 37 (0.5) | 28 (0.4) | 9 (0.1) | 16 (0.2) |
| Year 4 | 121 (1.7) | 74.0 (13.0) | 47 (0.7) | 38 (0.5) | 16 (0.2) | 20 (0.3) |
| Year 5 | 109 (1.5) | 75.0 (16.0) | 40 (0.6) | 33 (0.5) | 16 (0.2) | 20 (0.3) |
| Year 6 | 134 (1.9) | 77.0 (11.0) | 37 (0.5) | 48 (0.7) | 20 (0.3) | 29 (0.4) |
| Year 7 | 159 (2.2) | 76.0 (16.0) | 54 (0.8) | 41 (0.6) | 26 (0.4) | 38 (0.5) |
| Year 8 | 160 (2.2) | 74.0 (12.0) | 59 (0.8) | 44 (0.6) | 27 (0.4) | 30 (0.4) |
| Year 9 | 156 (2.2) | 75.0 (11.0) | 46 (0.6) | 41 (0.6) | 36 (0.5) | 33 (0.5) |
| Year 10 | 133 (1.9) | 72.0 (14.0) | 48 (0.7) | 35 (0.5) | 19 (0.3) | 31 (0.4) |
| Year 11 | 174 (2.4) | 72.0 (12.0) | 55 (0.8) | 46 (0.6) | 23 (0.3) | 50 (0.7) |
| Year 12 | 144 (2.0) | 73.0 (11.0) | 44 (0.6) | 40 (0.6) | 15 (0.2) | 45 (0.6) |
| Year 13 | 165 (2.3) | 71.0 (12.0) | 48 (0.7) | 47 (0.7) | 21 (0.3) | 49 (0.7) |
| Year 14 | 77 (1.1) | 73.0 (12.0) | 18 (0.3) | 23 (0.3) | 19 (0.3) | 17 (0.2) |

IQR – interquartile range (0.75-quantile minus 0.25-quantile); CVD, cardiovascular disease

**eTable 2.**  Comparisons between the ELSA participants who developed dementia and those who remained dementia free at the end of the average 10-year follow up period.

| **Baseline characteristics** | **Total sample*** | **Dementia by the end of follow-up** | | |  | |  |
| --- | --- | --- | --- | --- | --- | --- | --- |
|  | **N=7056** | **No** | **Yes** | **Test statistics** | | | |
|  |  | **N=6862 (97.3%)** | **N=194 (2.7%)** |  |  |  |  |
|  | Mean (SD) / n (%) | Mean (SD) / n (%) | Mean (SD) / n (%) | *t(df)/x2(df)* | | P | |
| Length of follow-up, years | 9.2 (2.0) | 9.2 (1.9) | 8.4 (2.2) | 5.45 (7054) | | <0.0001 | |
|  |  |  |  |  | |  | |
| Age (years) | 64.6 (9.4) | 64.3 (9.3) | 75.7 (8.7) | -16.82 (7054) | | <0.0001 | |
|  |  |  |  |  | |  | |
| Gender |  |  |  |  | |  | |
| Men | 3258 (46.2) | 3174 (46.3) | 84 (43.3) | 0.66 (1) | | 0.4154 | |
| Women | 3798 (53.8) | 3688 (53.7) | 110 (56.7) |  | |  | |
|  |  |  |  |  | |  | |
| APOEe4 present |  |  |  |  | |  | |
| Yes | 5280 (74.8) | 5164 (75.3) | 116 (59.8) | 3.76 (1) | | 0.0524 | |
| No | 1776 (25.2) | 1698 (24.7) | 78 (40.2) |  | |  | |
|  |  |  |  |  | |  | |
| Cause of mortality event |  |  |  |  | |  | |
| Any | 1205 (17.1) | 1106 (16.1) | 99 (51.0) | 338.1 (4) | | <0.0001 | |
| Cancer | 428 (6.1) | 413 (6.0) | 15 (7.7) |  | |  | |
| CVD | 358 (5.1) | 333 (4.9) | 25 (12.9) |  | |  | |
| Respiratory | 194 (2.7) | 182 (2.7) | 12 (6.2) |  | |  | |
| Other | 225 (3.2) | 178 (2.6) | 47 (24.2) |  | |  | |
|  |  |  |  |  | |  | |
| in % of all mortality events |  |  |  |  | |  | |
| Cancer | 35.5% | 37.3% | 15.2% |  | |  | |
| CVD | 29.7% | 30.1% | 25.3% |  | |  | |
| Respiratory | 16.1% | 16.5% | 12.1% |  | |  | |
| Other | 18.7% | 16.1% | 47.5% |  | |  | |

*excluding n=77 with unknown dementia status

**eTable 3.** An overview of the summary of full QC procedure employed in the ELSA study and how many variants and/or participants were lost at each step

| Quality Control steps in ELSA | | | |
| --- | --- | --- | --- |
| *Lost due to SNP-based QC* | | *n* | % |
|  | Missing SNPs (0.02) | 41614 | 1.87 |
|  | Autosomal SNPs | 48578 | 2.18 |
|  | MAF 0.01 | 759972 | 34.07 |
|  | Update rsids | 2284 | 0.10 |
|  | HWE (0.0001) | 6079 | 0.27 |
|  |  |  |  |
|  | *Total removed* | *858527* | *38.49* |
|  | *Total remaining* | *1372240* | *61.51* |
|  |  |  |  |
| *Lost due to Individual-based QC* | |  |  |
|  | Missingness (0.02) | 39 | 0.53 |
|  | Heterogeneity | 76 | 1.03 |
|  | Sex discordance | 5 | 0.07 |
|  | Ancestry outliers | 64 | 0.86 |
|  | Relatedness/Duplicates | 5 | 0.07 |
|  | Unique IDs are not present | 41 | 0.50 |
|  |  |  |  |
|  | *Total removed* | *229* | *3.09* |
|  | *Total remaining* | *7183* | *96.91* |

HWE, Hardy-Weinberg equilibrium; MAF, minor allele frequency; SNP, single nucleotide polymorphisms

**eTable 4.** Estimated the predictive accuracy (*R*^2^, p-value) for polygenic score for longevity

|  | | | ***p*-value threshold for Polygenic scores** | | | | | | | | | | | |
| --- | --- | --- | --- | --- | --- | --- | --- | --- | --- | --- | --- | --- | --- | --- |
|  |  |  | **0.001** | | **0.01** | | **0.05** | | **0.1** | | **0.3** | | **1** | |
|  |  |  | |  | |  | |  | |  | |  | |  |
| **Polygenic score_longevity_** | m | 2217 | | 17085 | | 75266 | | 145627 | | 417542 | | 1329210 | |  |
|  | *R*^2^ | 0.135 | | 0.037 | | 0.015 | | 0.011 | | 0.007 | | 0.003 | |  |
|  | *p-value* | 3.19×10^-77^ | | 6.66×10^-22^ | | 1.22×10^-09^ | | 1.39×10^-07^ | | 3.41×10^-05^ | | 0.006 | |  |

*M,* total number of independent markers in genotyping panel

**eTable 5.** Baseline sample characteristics of ELSA participants stratified by *APOE*-ε4 status

| **Baseline characteristics** | **Total sample** | ***APOE*-ε4 present** | | **Test statistics** | |
| --- | --- | --- | --- | --- | --- |
|  | **7131** | **No** | **Yes** |  |  |
|  |  | **5323 (74.6%)** | **1808 (25.4%)** |  |  |
|  | Mean (SD) / n (%) | Mean (SD) / n (%) | Mean (SD) / n (%) | *t*(df)/x^2^(df) | p |
| Length of follow-up, years | 9.1 (2.0) | 9.1 (2.0) | 9.1 (2.0) | -0.05 (7129) | 0.960 |
|  |  |  |  |  |  |
| Age (years) | 64.7 (9.5) | 64.8 (9.5) | 64.4 (9.4) | 1.57 (7129) | 0.117 |
| Gender |  |  |  |  |  |
| Men | 3292 (46.2) | 2479 (46.6) | 813 (45.0) | 1.4 (1) | 0.237 |
| Women | 3839 (53.8) | 2844 (53.4) | 995 (55.0) |  |  |
|  |  |  |  |  |  |
| Cause of mortality event |  |  |  |  |  |
| No (alive) | 5897 (82.7) | 4396 (82.6) | 1501 (83.0) | 9.58 (4) | 0.048 |
| Any | 1234 (17.3) | 927 (17.4) | 307 (17.0) |  |  |
| Cancer | 430 (6.0) | 325 (6.1) | 105 (5.8) |  |  |
| CVD | 367 (5.1) | 276 (5.2) | 91 (5.0) |  |  |
| Respiratory | 196 (2.7) | 160 (3.0) | 36 (2.0) |  |  |
| Other | 241 (3.4) | 166 (3.1) | 75 (4.1) |  |  |

**eTable 6.** Minimal Impact Size which can be found as statistically significant in the analytical sample with the power of 0.80 and type I error of 0.05

|  | APOE-ε4 (for 1/0) | | PGS_longevity_ (for 1-SD) | |
| --- | --- | --- | --- | --- |
|  | **Minimal Impact Size** | **Corresponding Hazard Ratio** | **Minimal Impact Size** | **Corresponding Hazard Ratio** |
| All-cause mortality | 0.18 | 1.20 | 0.08 | 0.93 |
|  |  |  |  |  |
| *Cause-specific mortality:* |  |  |  |  |
| Cancer | 0.15 | 1.16 | 0.13 | 0.88 |
| CVD | 0.34 | 1.40 | 0.15 | 0.86 |
| Respiratory | 0.51 | 1.67 | 0.20 | 0.82 |
| Other | 0.43 | 1.53 | 0.18 | 0.83 |

PGS, polygenic score for longevity; *APOE*-ε4, ε4 allele of the apolipoprotein E gene; HR, hazard ratio; CVD, cardiovascular disease

Minimal Impact Size is a Cox regression coefficient for the variables representing APOE-ε4 or PGS_longevity_. Corresponding Hazard Ratio is exponentiated Minimal Impact Size for APOE-ε4, and minus Minimal Impact Size for PGS_longevity_ as it is expected to have a protective impact on mortality.

Hazard ratio for PGS_longevity_ is expressed for 1 standard deviation change in PGS_longevity_;

Hazard ratio for *APOE*-ε4is for presence vs absence of specific APOE-ε4 allele

**eTable 7.** The statistics to show that assumptions for cox model were met

| **Covariates** | **All causes** | | | **Cancers** | | | **CVD** | | | **Respiratory** | | | **All other causes** | | |
| --- | --- | --- | --- | --- | --- | --- | --- | --- | --- | --- | --- | --- | --- | --- | --- |
|  | ***x*^2^** | **df** | **p** | ***x*^2^** | **df** | **p** | ***x*^2^** | **df** | **p** | ***x*^2^** | **df** | **p** | ***x*^2^** | **df** | **p** |
| Age (years) | 1.52 | 1 | 0.218 | 0.0198 | 1 | 0.888 | 1.29 | 1 | 0.256 | 5.63 | 1 | 0.018 | 0.51 | 1 | 0.475 |
| Gender | 3.58 | 1 | 0.059 | 0.0975 | 1 | 0.755 | 1.39 | 1 | 0.239 | 6.20 | 1 | 0.013 | 0.72 | 1 | 0.396 |
| *APOE*-ε4 | 0.33 | 1 | 0.563 | 0.5946 | 1 | 0.441 | 0.64 | 1 | 0.422 | 0.77 | 1 | 0.380 | 1.08 | 1 | 0.299 |
| PC 1 | 0.15 | 1 | 0.698 | 1.0791 | 1 | 0.299 | 0.17 | 1 | 0.679 | 0.07 | 1 | 0.786 | 2.33 | 1 | 0.127 |
| PC 2 | 0.16 | 1 | 0.712 | 0.0653 | 1 | 0.798 | 1.05 | 1 | 0.304 | 3.67 | 1 | 0.055 | 1.36 | 1 | 0.243 |
| PC 3 | 0.13 | 1 | 0.715 | 0.0056 | 1 | 0.941 | 0.14 | 1 | 0.705 | 2.27 | 1 | 0.132 | 0.41 | 1 | 0.522 |
| PC 4 | 0.48 | 1 | 0.490 | 0.0452 | 1 | 0.832 | 0.11 | 1 | 0.742 | 0.01 | 1 | 0.911 | 1.66 | 1 | 0.200 |
| PC 5 | 0.30 | 1 | 0.585 | 0.1340 | 1 | 0.714 | 0.00 | 1 | 0.946 | 2.63 | 1 | 0.105 | 0.31 | 1 | 0.575 |
| PC 6 | 0.21 | 1 | 0.643 | 1.6595 | 1 | 0.198 | 5.31 | 1 | 0.021 | 0.22 | 1 | 0.637 | 0.90 | 1 | 0.344 |
| PC 7 | 0.32 | 1 | 0.571 | 2.8420 | 1 | 0.092 | 0.05 | 1 | 0.828 | 0.00 | 1 | 0.965 | 2.37 | 1 | 0.124 |
| PC 8 | 0.34 | 1 | 0.560 | 0.0224 | 1 | 0.881 | 0.64 | 1 | 0.425 | 0.09 | 1 | 0.769 | 0.00 | 1 | 0.988 |
| PC 9 | 0.37 | 1 | 0.544 | 1.5078 | 1 | 0.219 | 0.28 | 1 | 0.599 | 5.94 | 1 | 0.015 | 0.08 | 1 | 0.775 |
| PC 10 | 0.15 | 1 | 0.702 | 0.9214 | 1 | 0.337 | 0.08 | 1 | 0.775 | 0.44 | 1 | 0.506 | 1.73 | 1 | 0.188 |
| PGC_longevity_ | 4.58 | 1 | 0.032 | 2.4660 | 1 | 0.116 | 2.78 | 1 | 0.095 | 0.01 | 1 | 0.935 | 0.12 | 1 | 0.725 |
| **GLOBAL** | **12.84** | **14** | **0.539** | **11.3730** | **14** | **0.656** | **14.76** | **14** | **0.395** | **26.07** | **14** | **0.025** | **13.29** | **14** | **0.504** |

*APOE*-ε4, ε4 allele of the apolipoprotein E gene; PGS, Polygenic score, PC, principal component; CVD, cardiovascular disease; df, degrees of freedom; All covariates and the global test (for the overall model) passed the test as p-values > 0.05.

**eTable 8.** Baseline sample characteristics of ELSA participants stratified by age

| **Baseline characteristics** | **Total sample** | **Baseline age** | |  | |
| --- | --- | --- | --- | --- | --- |
|  | **7131** | **50-75 years old** | **>75 years old** | **Test statistics** | |
|  |  | **6057 (84.9%)** | **1074 (15.1%)** |  |  |
|  | Mean (SD) / n (%) | Mean (SD) / n (%) | Mean (SD) / n (%) | *t*(df)/x^2^(df) | p |
| Length of follow-up, years | 9.1 (2.0) | 9.4 (1.5) | 7.5 (3.0) | 32.48 (7129) | <0.001 |
|  |  |  |  |  |  |
| Age (years) | 64.7 (9.5) | 61.8 (6.9) | 81 (4.2) | -88.77 (7129) | <0.001 |
|  |  |  |  |  |  |
| Gender |  |  |  |  |  |
| Men | 3292 (46.2) | 2824 (46.6) | 468 (43.6) | 3.41 (1) | 0.0648 |
| Women | 3839 (53.8) | 3233 (53.4) | 606 (56.4) |  |  |
|  |  |  |  |  |  |
| Mortality event |  |  |  |  |  |
| None (alive) | 5897 (82.7) | 5422 (89.5) | 475 (44.2) | 1467.56 (4) | <0.001 |
| Yes, from which cause: | 1234 (17.3) | 635 (10.5) | 599 (55.8) |  |  |
| Cancer | 430 (34.8) | 297 (46.8) | 133 (22.2) |  |  |
| CVD | 367 (29.7) | 155 (24.4) | 212 (35.4) |  |  |
| Respiratory | 196 (15.9) | 82 (12.9) | 114 (19.0) |  |  |
| Other | 241 (19.5) | 101 (15.9) | 140 (23.4) |  |  |

**eTable 9.**  Comparisons of the ELSA participants by gender.

| **Baseline characteristics** | **Total sample** | **Sex** | |  |  |
| --- | --- | --- | --- | --- | --- |
|  | **N=7131** | **Male** | **Female** | **Test statistics** | |
|  |  | **N=3292 (46.2%)** | **N=3839 (53.8%)** |  |  |
|  | Mean (SD) / n (%) | Mean (SD)/ n (%) | Mean (SD) / n (%) | *t*(df)/x^2^(df) | P |
| Length of follow-up, years | 9.1(2) | 9(2.2) | 9.3(1.7) | -7.15(7129) | <0.001 |
|  |  |  |  |  |  |
| Age (years) | 64.7(9.5) | 64.6(9.2) | 64.8(9.7) | -0.9(7129) | 0.3671 |
| Gender |  |  |  |  |  |
| Men | 3292(46.2) | 3292(100) | 0(0) | 0.0 (1) | <0.001 |
| Women | 3839(53.8) | 0(0) | 3839(100) |  |  |
|  |  |  |  |  |  |
| Accumulated wealth |  |  |  |  |  |
| High | 2428(34.9) | 1199(37.3) | 1229(32.7) | 20.33(2) | <0.001 |
| Intermediate | 2136(30.7) | 982(30.6) | 1154(30.7) |  |  |
| Low | 2400(34.5) | 1030(32.1) | 1370(36.5) |  |  |
|  |  |  |  |  |  |
| Years of education | 14(3.8) | 15(3.9) | 14(3.7) | 10.33(7129) | <0.001 |
|  |  |  |  |  |  |
| Smoking status |  |  |  |  |  |
| Non-smoker | 5951(83.8) | 2769(84.5) | 3182(83.2) | 2.3(1) | 0.1295 |
| Smoker | 1152(16.2) | 508(15.5) | 644(16.8) |  |  |
|  |  |  |  |  |  |
| Severe depressive symptom present |  |  |  |  |  |
| No | 6115(86) | 2957(90.1) | 3158(82.4) | 87.19(1) | <0.001 |
| Yes | 998(14) | 324(9.9) | 674(17.6) |  |  |
|  |  |  |  |  |  |
| Cause of mortality event |  |  |  |  |  |
| No (alive) | 5897(82.7) | 2618(79.5) | 3279(85.4) | 49.86(4) | <0.001 |
| Any | 1234(17.3) | 674(20.5) | 560(14.6) |  |  |
| Cancer | 430(6) | 248(7.5) | 182(4.7) |  |  |
| CDV | 367(5.1) | 194(5.9) | 173(4.5) |  |  |
| Respiratory | 196(2.7) | 115(3.5) | 81(2.1) |  |  |
| Other | 241(3.4) | 117(3.6) | 124(3.2) |  |  |

**eTable 10.** Cox regression analyses highlighting associations between PGS_longevity_ and *APOE*-ε4 and risk for cause-specific mortality during the 10-year follow-up period; the analyses are limited to adults who are aged ≤75 years old

| **Models** | **Specific causes of mortality** | | | | |
| --- | --- | --- | --- | --- | --- |
|  | **All** | **Cancer** | **CVD** | **Respiratory** | **Other** |
| **Cause-specific Cox model** | **HR (95%CI), p** | **HR (95%CI), p** | **HR (95%CI), p** | **HR (95%CI), p** | **HR (95%CI), p** |
| PGS _longevity_ | 0.93 (0.86, 1.0), 0.080 | 0.93 (0.82,1.04),  0.208 | 0.89 (0.76, 1.05), 0.170 | 1.08 (0.87, 1.33), 0.499 | 0.89 (0.72, 1.09), 0.245 |
| *APOE*-ε4 | 1.04 (0.87,1.25), 0.663 | 0.88 (0.67,1.16),  0.381 | 1.20 (0.84, 1.72), 0.303 | 0.70 (0.40, 1.23), 0.213 | 1.66 (1.10, 2.51), 0.015 |
|  |  |  |  |  |  |
| **Fine-Gray model** | **HR (95%CI), p** | **HR (95%CI), p** | **HR (95%CI), p** | **HR (95%CI), p** | **HR (95%CI), p** |
| PGS _longevity_ | - | 0.93 (0.83, 1.04), 0.190 | 0.89 (0.76, 1.05), 0.180 | 1.08 (0.87, 1.34), 0.490 | 0.89 (0.70, 1.12), 0.310 |
| *APOE*-ε4 | - | 0.87 (0.66, 1.15), 0.320 | 1.18 (0.82, 1.70), 0.370 | 0.68 (0.39, 1.20), 0.180 | 1.64 (1.08, 2.49), 0.019 |

PGS, polygenic score for longevity; *APOE*-ε4, ε4 allele of the apolipoprotein E gene; HR, hazard ratio; CI, confidence intervals; CVD, cardiovascular disease

**eTable 11.** Mediation analysis for the impact of the *APOE*-ε4 on specific causes of mortality in the following 10 years mediated by a dementia diagnosis; the analyses were restricted to the participants who were ≤75 years old at baseline.

| **Causes of mortality** | **All** | **Cancer** | **CVD** | **Respiratory** | **Other** |
| --- | --- | --- | --- | --- | --- |
|  | **HR (95%CI),**  **p-value** | **HR (95%CI),**  **p-value** | **HR (95%CI), p-value** | **HR (95%CI),**  **p-value** | **HR (95%CI), p-value** |
| *Direct effect* | 0.96 (0.80, 1.16), 0.680 | 0.87 (0.66, 1.15), 0.323 | 1.15 (0.80, 1.66), 0.456 | 0.69 (0.39, 1.22), 0.207 | 1.29 (0.82, 2.02), 0.272 |
|  |  |  |  |  |  |
| *Indirect effect* | 1.03 (0.99, 1.06), 0.058 | 1.01 (0.98, 1.03), 0.659 | 1.03 (0.98, 1.07), 0.251 | 1.01 (0.96, 1.06), 0.689 | 1.12 (0.99, 1.25), 0.064 |
|  |  |  |  |  |  |
| *Total effect* | 0.99 (0.82, 1.19), 0.909 | 0.87 (0.66, 1.15), 0.338 | 1.18 (0.82, 1.69), 0.373 | 0.70 (0.40, 1.23), 0.215 | 1.43 (0.92, 2.24), 0.112 |
|  |  |  |  |  |  |
| Percent mediated by dementia | -2.59 | -4.0% | 16% | -2.0% | 34% |

APOE-ε4, ε4 allele of the apolipoprotein E gene; HR, hazard ratio; CI, confidence intervals; CVD, cardiovascular diseas

**eTable 12.** Cox regression analyses highlighting associations between PGS_longevity_ and *APOE*-ε4 and risk for cause-specific mortality during the 10-year follow-up period; the analyses are limited to adults who were aged >75 years old at baseline.

|  | PGS _longevity_ | | | | | | | | |
| --- | --- | --- | --- | --- | --- | --- | --- | --- | --- |
| **Models** | *APOE*-ε4 | | **Cancer** | | **CVD** | | **Respiratory** | | **Other** |
| **Cause-specific Cox model** | **HR (95%CI), p** | | **HR (95%CI), p** | | **HR (95%CI), p** | | **HR (95%CI), p** | | **HR (95%CI), p** |
| PGS _longevity_ | 0.93 (0.85, 1.01), 0.069 | | 1.04 (0.88, 1.24), 0.628 | | 1.00 (0.88, 1.15), 0.949 | | 0.85 (0.70,1.03), 0.086 | | 0.76 (0.63, 0.90), 0.002 |
| *APOE*-ε4 | 0.97 (0.780, 1.17), 0.735 | | 1.23 (0.84, 1.81), 0.283 | | 0.90 (0.65, 1.25), 0.521 | | 0.70 (0.43, 1.13), 0.138 | | 1.06 (0.73, 1.56), 0.744 |
|  | |  | |  | |  | |  | |
| **Fine-Gray model** | **HR (95%CI), p** | | **HR (95%CI), p** | | **HR (95%CI), p** | | **HR (95%CI), p** | | **HR (95%CI), p** |
| PGS _longevity_ | - | | 1.06 (0.89, 1.27), 0.48 | | 1.03 (0.899, 1.17), 0.69 | | 0.86 (0.72, 1.04), 0.11 | | 0.76 (0.62, 0.92), 0.0051 |
| *APOE*-ε4 | - | | 1.26 (0.85, 1.87), 0.24 | | 0.93 (0.671, 1.29), 0.67 | | 0.72 (0.44, 1.18), 0.19 | | 1.11 (0.75,1.63), 0.61 |

PGS, polygenic score for longevity; *APOE*-ε4, ε4 allele of the apolipoprotein E gene; HR, hazard ratio; CI, confidence intervals; CVD, cardiovascular disease

**eTable 13.** Mediation analysis for the impact of the *APOE*-ε4 on specific causes of mortality in the following 10 years mediated by a dementia diagnosis; the analyses were restricted to the participants who were aged >75 years old at baseline.

| **Causes of mortality** | **All** | **Cancer** | **CVD** | **Respiratory** | **Other** |
| --- | --- | --- | --- | --- | --- |
|  | **HR (95%CI),**  **p-value** | **HR (95%CI),**  **p-value** | **HR (95%CI), p-value** | **HR (95%CI),**  **p-value** | **HR (95%CI), p-value** |
| *Direct effect* | 1.01 (0.82, 1.23), 0.986 | 1.30 (0.88, 1.94), 0.191 | 1.01 (0.71, 1.41), 0.976 | 0.70 (0.42, 1.17), 0.172 | 1.00 (0.64, 1.57), 0.991 |
|  |  |  |  |  |  |
| *Indirect effect* | 1.00 (0.99, 1.01), 0.732 | 0.99 (0.97, 1.01), 0.198 | 0.98 (0.97, 1.00), 0.117 | 1.00 (0.97, 1.04), 0.863 | 1.03 (0.98, 1.09), 0.190 |
|  |  |  |  |  |  |
| *Total effect* | 1.00 (0.82, 1.22), 0.999 | 1.29 (0.87, 1.91), 0.211 | 0.99 (0.70, 1.39), 0.954 | 0.70 (0.42, 1.16), 0.167 | 1.03 (0.67, 1.59), 0.891 |
|  |  |  |  |  |  |
| Percent mediated by dementia | 18.82 | -6.0% | 1.53 | -0.7% | 1.08 |

APOE-ε4, ε4 allele of the apolipoprotein E gene; HR, hazard ratio; CI, confidence intervals; CVD, cardiovascular disease

**eTable 14.** Cox regression analyses highlighting associations between PGS_longevity_ and *APOE*-ε4 and risk for cause-specific mortality during the 10-year follow-up period stratified by gender.

|  | **Specific causes of mortality** | | | | | | | | | | | | | | |
| --- | --- | --- | --- | --- | --- | --- | --- | --- | --- | --- | --- | --- | --- | --- | --- |
|  | **All** | | | **Cancer** | | | **CVD** | | | **Respiratory** | | | **Other** | | |
| **Cox model** | **HR** | **95%CI** | **P value** | **HR** | **95%CI** | **P value** | **HR** | **95%CI** | **P value** | **HR** | **95%CI** | **P value** | **HR** | **95%CI** | **P value** |
|  |  |  |  |  |  |  |  |  |  |  |  |  |  |  |  |
| **MEN** |  |  |  |  |  |  |  |  |  |  |  |  |  |  |  |
| PGS _longevity_ | 1.01 | 0.85-1.21 | 0.879 | 1.24 | 0.94-1.64 | 0.129 | 0.86 | 0.61-1.22 | 0.402 | 0.71 | 0.44-1.15 | 0.163 | 1.15 | 0.76-1.74 | 0.505 |
| *APOE*-ε4 | 0.94 | 0.87-1.02 | 0.132 | 1.03 | 0.91-1.16 | 0.638 | 0.87 | 0.75-1.01 | 0.062 | 0.98 | 0.82-1.18 | 0.867 | 0.83 | 0.68-1.00 | 0.050 |
|  |  |  |  |  |  |  |  |  |  |  |  |  |  |  |  |
| **WOMEN** |  |  |  |  |  |  |  |  |  |  |  |  |  |  |  |
| PGS _longevity_ | 0.98 | 0.81-1.19 | 0.843 | 0.67 | 0.46-0.98 | 0.035 | 1.17 | 0.84-1.64 | 0.351 | 0.70 | 0.39-1.24 | 0.213 | 1.44 | 0.98-2.10 | 0.058 |
| *APOE*-ε4 | 0.91 | 0.83-0.99 | 0.030 | 0.85 | 0.73-0.99 | 0.039 | 1.07 | 0.93-1.25 | 0.343 | 0.89 | 0.70-1.13 | 0.332 | 0.79 | 0.65-0.96 | 0.014 |
|  |  |  |  |  |  |  |  |  |  |  |  |  |  |  |  |
|  |  |  |  |  |  |  |  |  |  |  |  |  |  |  |  |
|  | **All** | | | **Cancer** | | | **CVD** | | | **Respiratory** | | | **Other** | | |
| **Fine-Gray model** | **HR** | **95%CI** | **P value** | **HR** | **95%CI** | **P value** | **HR** | **95%CI** | **P value** | **HR** | **95%CI** | **P value** | **HR** | **95%CI** | **P value** |
| **MEN** |  |  |  |  |  |  |  |  |  |  |  |  |  |  |  |
| PGS _longevity_ |  |  |  | 1.27 | 0.96-1.68 | 0.096 | 0.85 | 0.60-1.21 | 0.360 | 0.73 | 0.45-1.19 | 0.200 | 1.17 | 0.77-1.78 | 0.460 |
| *APOE*-ε4 |  |  |  | 1.04 | 0.92-1.17 | 0.530 | 0.88 | 0.76-1.01 | 0.065 | 0.99 | 0.82-1.19 | 0.900 | 0.82 | 0.66-1.01 | 0.062 |
|  |  |  |  |  |  |  |  |  |  |  |  |  |  |  |  |
| **WOMEN** |  |  |  |  |  |  |  |  |  |  |  |  |  |  |  |
| PGS _longevity_ |  |  |  | 0.67 | 0.46-0.97 | 0.033 | 1.24 | 0.88-1.77 | 0.220 | 0.66 | 0.37-1.18 | 0.160 | 1.43 | 0.97-2.11 | 0.068 |
| *APOE*-ε4 |  |  |  | 0.85 | 0.74-0.99 | 0.037 | 1.11 | 0.95-1.30 | 0.190 | 0.90 | 0.72-1.13 | 0.360 | 0.80 | 0.64-0.99 | 0.035 |

PGS, polygenic score for longevity; *APOE*-ε4, ε4 allele of the apolipoprotein E gene; HR, hazard ratio; CI, confidence intervals; CVD, cardiovascular disease

**eTable 15.** Mediation analysis for the impact of the *APOE*-ε4 on specific causes of mortality in the following 10 years mediated by a dementia diagnosis stratified by gender.

| **Causes of mortality** | **All** | **Cancer** | **CVD** | **Respiratory** | **Other** |
| --- | --- | --- | --- | --- | --- |
|  | **HR (95%CI), p-value** | **HR (95%CI), p-value** | **HR (95%CI), p-value** | **HR (95%CI), p-value** | **HR (95%CI), p-value** |
| **MEN:** |  |  |  |  |  |
| *Direct effect* | 0.97 (0.81, 1.17), 0.774 | 1.22 (0.92, 1.62), 0.169 | 0.85 (0.59, 1.21), 0.364 | 0.71 (0.44, 1.16), 0.176 | 0.97 (0.62, 1.52), 0.896 |
|  |  |  |  |  |  |
| *Indirect effect* | 1.01 (0.99, 1.03), 0.426 | 1.00 (0.97, 1.03), 0.894 | 1.00 (0.96, 1.04), 0.982 | 1.00 (0.95, 1.05), 0.886 | 1.07 (0.98, 1.16), 0.156 |
|  |  |  |  |  |  |
| *Total effect* | 0.98 (0.82, 1.18), 0.850 | 1.22 (0.92, 1.61), 0.170 | 0.85 (0.59, 1.21), 0.358 | 0.71 (0.44, 1.15), 0.165 | 1.03 (0.67, 1.6), 0.879 |
|  |  |  |  |  |  |
| Percent mediated by dementia | -53% | -1% | 0% | 1% | 0% |
|  |  |  |  |  |  |
| **WOMEN:** |  |  |  |  |  |
| *Direct effect* | 0.96 (0.79, 1.18), 0.724 | 0.69 (0.47, 1.00), 0.052 | 1.31 (0.93, 1.85), 0.125 | 0.7 (0.39, 1.28), 0.248 | 1.24 (0.8, 1.91), 0.337 |
|  |  |  |  |  |  |
| *Indirect effect* | 1.02 (0.99, 1.04), 0.266 | 0.98 (0.94, 1.02), 0.401 | 0.98 (0.96, 1.01), 0.140 | 1.02 (0.94, 1.10), 0.642 | 1.09 (1.00, 1.20), 0.061 |
|  |  |  |  |  |  |
| *Total effect* | 0.98 (0.8, 1.19), 0.833 | 0.68 (0.46, 0.98), 0.040 | 1.29 (0.91, 1.81), 0.150 | 0.71 (0.4, 1.28), 0.257 | 1.35 (0.89, 2.04), 0.156 |
|  |  |  |  |  |  |
| Percent mediated by dementia | -7% | 4% | -9% | -4% | 32% |

APOE-ε4, ε4 allele of the apolipoprotein E gene; HR, hazard ratio; CI, confidence intervals; CVD, cardiovascular disease

**eTable 16.** Cox regression analyses highlighting associations between dementia diagnosis and risk for cause-specific mortality during the 10-year follow-up period.

| **Causes of mortality** | **All** | **Cancer** | **CVD** | **Respiratory** | **Other** |
| --- | --- | --- | --- | --- | --- |
|  | **HR (95%CI), p** | **HR (95%CI), p** | **HR (95%CI), p** | **HR (95%CI), p** | **HR (95%CI), p** |
| ***Entire sample*** | 1.1 (0.84, 1.45), 0.49 | 0.55 (0.27, 1.13), 0.10 | 0.84 (0.5, 1.41), 0.50 | 0.73 (0.34, 1.59), 0.43 | 2.83 (1.82, 4.4), 0.001 |
|  |  |  |  |  |  |
| ***Men*** | 1.41 (0.95, 2.1), 0.08 | 0.67 (0.24, 1.83), 0.43 | 1.05 (0.48, 2.29), 0.91 | 0.95 (0.34, 2.66), 0.92 | 4.09 (2.17, 7.7), 0.001 |
|  |  |  |  |  |  |
| ***Women*** | 0.91 (0.62, 1.34), 0.64 | 0.47 (0.17, 1.3), 0.14 | 0.74 (0.37, 1.5), 0.40 | 0.58 (0.18, 1.88), 0.36 | 2.24 (1.21, 4.15), 0.01 |

HR, hazard ratio; CI, confidence intervals; CVD, cardiovascular disease
